# YTHDF2 facilitates *UBXN1* mRNA decay by recognizing METTL3-mediated m^6^A modification to activate NF-κB and promote the malignant progression of glioma

**DOI:** 10.1186/s13045-021-01124-z

**Published:** 2021-07-10

**Authors:** Rui-Chao Chai, Yu-Zhou Chang, Xin Chang, Bo Pang, Song Yuan An, Ke-Nan Zhang, Yuan-Hao Chang, Tao Jiang, Yong-Zhi Wang

**Affiliations:** 1 Department of Molecular Neuropathology, Department of Neuropathology, Beijing Neurosurgical Institute, Capital Medical University, No. 119 Nan Si Huan Xi Road, Fengtai District, Beijing, 100050 China; 2 Department of Neurosurgery, Beijing Tiantan Hospital, Capital Medical University, No. 119 Nan Si Huan Xi Road, Fengtai District, Beijing, 100050 China; 3 Department of Neurosurgery, Sanbo Brain Hospital, Capital Medical University, Beijing, China; 4 Chinese Glioma Genome Atlas Network (CGGA), Beijing, China

**Keywords:** *N*6,2′-*O*-Dimethyladenosine, Glioblastoma, YTHDF2, METTL3, NF-κB activation

## Abstract

**Background:**

The prognosis for diffuse gliomas is very poor and the mechanism underlying their malignant progression remains unclear. Here, we aimed to elucidate the role and mechanism of the RNA *N*6,2′-*O*-dimethyladenosine (m^6^A) reader, YTH *N*6-methyladenosine RNA binding protein 2 (YTHDF2), in regulating the malignant progression of gliomas.

**Methods:**

YTHDF2 mRNA levels and functions were assessed using several independent datasets. Western blotting, quantitative polymerase chain reaction, and immunohistochemistry were used to evaluate the expression levels of YTHDF2 and other molecules in human and mouse tumor tissues and cells. Knockdown and overexpression were used to evaluate the effects of YTHDF2, methyltransferase-like 3 (METTL3), and UBX domain protein 1 (UBXN1) on glioma malignancy in cell and orthotopic xenograft models. RNA immunoprecipitation (RIP), methylated RIP, and RNA stability experiments were performed to study the mechanisms underlying the oncogenic role of YTHDF2.

**Results:**

YTHDF2 expression was positively associated with a higher malignant grade and molecular subtype of glioma and poorer prognosis. YTHDF2 promoted the malignant progression of gliomas in both in vitro and in vivo models. Mechanistically, YTHDF2 accelerated *UBXN1* mRNA degradation via METTL3-mediated m^6^A, which, in turn, promoted NF-κB activation. We further revealed that UBXN1 overexpression attenuated the oncogenic effect of YTHDF2 overexpression and was associated with better survival in patients with elevated YTHDF2 expression.

**Conclusions:**

Our findings confirmed that YTHDF2 promotes the malignant progression of gliomas and revealed important insight into the upstream regulatory mechanism of NF-κB activation via UBXN1 with a primary focus on m^6^A modification.

**Supplementary Information:**

The online version contains supplementary material available at 10.1186/s13045-021-01124-z.

## Background

Adult diffuse gliomas are classified into grades II–IV based on histological features. They can also be divided into different molecular subgroups according to isocitrate dehydrogenase [NADP( +)] (*IDH*)-mutant and 1p/19q co-deletion status according to the latest World Health Organization classification of central nervous system tumors [1, 2]. The prognosis of gliomas is still very poor, especially for *IDH*-wild-type glioblastoma (GBM, WHO grade IV). The median overall survival of *IDH*-wild-type GBM is only about 14.4–15.2 months, which is attributed to the high proliferative and invasive nature of this diffuse glioma subtype [2–4]. Lower-grade gliomas (WHO grade II/III) often develop into secondary glioblastomas over several years of malignant progression [5, 6]. Hence, understanding the molecular mechanisms that underlie the malignant progression of diffuse gliomas is urgently needed.

Recent advances in understanding RNA *N*6,2′-*O*-dimethyladenosine (m^6^A) modification have provided new insights into the molecular mechanism of malignant progression of diffuse gliomas [7–9]. Similar to DNA and protein modification, m^6^A is dynamically regulated by methyltransferases (‘writers’) and demethylases (‘erasers’) [10, 11]. m^6^A can influence the entire RNA life cycle, including alternative polyadenylation, pre-RNA processing, RNA export from the nucleus to the cytoplasm, RNA translation, and RNA decay, through binding with different ‘readers’ of m^6^A [12–14]. We have studied the expression levels and potential functions of widely recognized m^6^A writers (METTL3, METTL14, WTAP, KIAA1429, RBM15, and ZC3H13), erasers (FTO and ALKBH5), and readers (YTHDC1, YTHDC2, YTHDF1, YTHDF2, YTHDF3, and HNRNPC) in diffuse gliomas and have revealed that the expression level of YTHDF2 (YTH *N*6-methyladenosine RNA binding protein 2) is significantly correlated with increased malignancy [9]. However, the specific effects and underlying mechanism of up-regulated YTHDF2 expression on malignant progression are less well understood.

YTHDF2 destabilizes m^6^A-modified RNA via serval different mechanisms, including de-adenylation by the CCR4–NOT complex, and endoribonucleolytic cleavage by HRSP12-RNase P/MRP [15–17]. YTHDF2 promotes GBM stem cell growth by enhancing MYC stability [18] and is involved in GBM tumorigenesis by promoting cholesterol dysregulation [19]. These findings indicate that YTHDF2 may be involved in tumorigenesis and malignant progression of glioma through multiple pathways. Emerging evidence shows that activation of the NF-kB signaling pathway plays a core role in promoting the malignant progression of glioma [3, 20, 21]. However, whether YTHDF2 can modulate NF-kB activation in gliomas through m^6^A modification remains unclear.

Here, we investigated the expression levels of YTHDF2 mRNA and protein in gliomas and observed that YTHDF2 expression was positively correlated with increased glioma malignancy. Elevated YTHDF2 expression was associated with decreased survival of all diffuse glioma and GBM patients in both the Chinese Glioma Genome Atlas (CGGA) and The Cancer Genome Atlas (TCGA) datasets. We demonstrate that YTHDF2 can promote cell proliferation and migration in several glioma models. Additionally, we show that elevated YTHDF2 can promote nuclear factor kappa B (NF-κB) activation by suppressing UBX domain protein 1 (UBXN1) expression. Mechanistically, we show that YTHDF2 can accelerate *UBXN1* mRNA degradation by recognizing m^6^A modification mediated by the m^6^A writer, METTL3. Finally, we also validate the role of YTHDF2 in promoting tumor progression and activating NF-κB signaling in orthotopic xenograft models.

## Methods

### Samples and databases

We obtained transcriptional and clinical data from the CGGA official website (http://www.cgga.org.cn/) and the TCGA database (https://tcga-data.nci.nih.gov/tcga/tcgaDownload.jsp) [22, 23]. The clinical samples used to study protein levels were collected from the CGGA tissue bank and were supervised by the Beijing Tiantan Hospital institutional review board (KY2019-143-02).

### Cell culture

All cells were cultured as previously reported [13, 24]. Briefly, human astrocytes (HA; cat. no. 1800; ScienCell Research Laboratories, Inc., San Diego, CA, USA) were cultured in astrocyte medium (ScienCell). The cell lines, H4, LN229, and U87, were purchased from the Institute of Basic Medicine, Chinese Academy of Medical Sciences and Union Medical College (Beijing, China) and cultured in Dulbecco’s modified Eagle’s medium (DMEM) (Gibco, NY, USA) supplemented with 10% fetal bovine serum (FBS; Gibco) and 1% penicillin–streptomycin (Gibco). All cell lines were previously authenticated by short tandem repeat analysis. The GBM patient-derived cell line, N33, was developed by CGGA and was cultured in DMEM/F12 (Gibco) supplemented with 10% FBS (Gibco) and 1% penicillin–streptomycin (Gibco) [25]. All cells were cultured at 37 °C with 5% CO_2_.

### Cell proliferation and Transwell migration assays

Cell proliferation was measured using the Cell Counting Kit-8 (CCK-8, Dojindo Laboratories, Kumamoto, Japan). Specifically, 1.5 × 10^3^ cells/well were seeded into six pairs of duplicate wells of a 96-well plate and incubated at 37 °C for 1–6 days. At the predesigned time point, the medium for six-repeated wells was changed, and 10 μl CCK-8 reagent was added to the 100 μl fresh culture medium. Absorbance was detected daily at 450 nm for 1–6 days.

For migration assays, GBM cells (1 × 10^5^) in serum-free DMEM were added to the Transwell upper chamber, with the lower chamber containing DMEM with 10% FBS. After incubation for 6 h at 37 °C, the migrated cells present on the underside of the Transwell membrane were stained with crystal violet and counted under a microscope (Zeiss, Germany).

### RNA extraction and quantitative polymerase chain reaction (qPCR) analysis

We extracted total RNA was using TRIzol reagent (Invitrogen, USA) following the manufacturer’s protocol and quantified samples with a NanoDrop 2000. cDNA was generated from 1 µg RNA and real-time qPCR was performed on a 7500 Fast Real-Time PCR system to determine target RNA levels. The primer sequences used are listed in Additional file 1: Table S1.

### Western blotting

Western blotting was performed as previously described [24]. After washing with ice-cold PBS three times, cells were lysed in ice-cold lysis buffer [150 mM NaCl, 0.1% (w/v) NP-40, 50 mM Tris (pH 8.0), 0.5% (w/v) sodium deoxycholate, 1% (w/v) SDS, 1 mM DTT, 0.1 mM PMSF]. A Bicinchoninic Acid Protein Assay kit (Thermo Fisher Scientific, Inc.) was used to measure total protein content. The proteins were boiled and then separated by 10% sodium dodecyl sulfate polyacrylamide gel electrophoresis (SDS-PAGE). After membrane transfer, membranes were incubated with primary antibodies targeting YTHDF2 (Abcam, 1:2000 dilution), METTL3 (Abcam, 1:2000 dilution), NF-κB p65 (CST, 1:1000 dilution), phospho-NF-κB p65 (Abcam, 1:1000 dilution), or GAPDH (1:4000, Proteintech) in blocking solution overnight at 4 ℃. Secondary goat anti-rabbit (1:2000; ZSGB-Bio) or goat anti-mouse (1:2000; ZSGB-Bio) antibodies conjugated with a horseradish peroxidase were incubated for 1 h at room temperature. A chemiluminescent Western blot detection kit (GE, USA) was used to detect target proteins.

### Transient transfection with siRNA

Scrambled siRNA and YTHDF2 siRNA were purchased from GenePharma (Suzhou, China). The siRNAs were transfected into cells using PolyPlus-transfection according to the manufacturer’s instructions (POLYSCIENCES, PA, USA). The growth medium was changed 8 h after transfection. Target RNAs and proteins were measured by qRT-PCR and Western blotting, respectively, 48 h after transfection. Two non-overlapping siRNAs that effectively suppressed target gene expression were selected for subsequent experiments. The sequences of siRNAs used are presented in Additional file 1: Table S2.

### Lentivirus vector infection

Lentiviruses expressing YTHDF2, METTL3, UBXN1, METTL3-shRNA (target sequence: gc CTT AAC ATT GCC CAC TGA T), YTHDF2-shRNA (target sequence 1: TGG ATA TAG TAG CAA TTA T; target sequence 2: ACA GGC AAG GCC CAA TAA T), scrambled shRNA, or empty vector were purchased from Beijing Syngentech Company and Shanghai Genechem Company.

U87, LN229, and N33 cells were selected to establish stable YTHDF2 overexpression cell lines. U87 cells were used to establish METTL3 knockdown and overexpression cell lines. UBXN1 was overexpressed in YTHDF2-overexpressing U87 cells. Transfection was performed as previously reported [25]. Briefly, 5 × 10^4^ cells were plated into a 6-well plate and transfected with the indicated lentivirus using HitransG P (Genechem) in accordance with the manufacturer’s instructions. Infected cells were selected using 2 μg/ml puromycin (MCE, USA) for ≥ 1 week, and the transfection efficiency was determined by RT-qPCR and Western blotting analysis.

### Methylated RNA immunoprecipitation

After extracting total RNA (200 µg) using TRIzol reagent, ribosomal RNA was removed using a RiboMinus™ Eukaryote Kit v2 (A15020, Invitrogen). Then, the RNA was sheared into approximately 100-nucleotide fragments using RNA Fragmentation Reagents (AM8740, Invitrogen). Next, a portion of the RNA solution was stored at − 80 °C as input for RNA sequencing or PCR. An anti-m^6^A antibody (Abcam) was incubated with RNA for 1 h at 4 °C. Prewashed Pierce™ Protein A/G Magnetic Beads (88803, Thermo Scientific) were mixed with the antibody-treated RNA in immunoprecipitation buffer at 4 °C overnight. Finally, the methylated RNA was purified for sequencing or PCR.

### RNA immunoprecipitation (RIP) assays

RIP assays were performed using the Magna RIP RNA-Binding Protein Immunoprecipitation Kit (Millipore, 17-700) according to the manufacturer’s instructions. IP lysis buffer was used to lyse U87 cells and the cell lysate was divided into anti-SNRNP70, anti-METTL3, anti-YTHDF2, anti-IgG, and input samples. The cell lysates were incubated with magnetic beads coated with 5 µg of specific antibodies overnight at 4 °C. After washing, the lysates were digested with Proteinase K, and the RNA bound to immunoprecipitated proteins was purified. qPCR was performed to measure the target RNA levels.

### RNA stability assays

Cells were seeded in 6-well plates, and siRNAs were used to knockdown specific expression for 2 days if necessary. Then, cells were treated with Actinomycin D (CST, 5 µg/ml) for 0, 1, 2, and 4 h or longer. RNA with added bacterial 16 s RNA was then extracted by TRIzol. The target RNA was measured by qPCR, and the bacterial 16 s RNA was used as a reference.

### Orthotopic xenograft models

All experiments were approved by Beijing Neurosurgical Institute following the U.K. Animals (Scientific Procedures) Act, 1986 and associated guidelines. Five-week-old female BALB/c nude mice (Charles Rivers, Beijing, China) were selected for the experiments. U87 cells (5 × 10^5^) transfected with an empty vector, YTHDF2 overexpression, or METTL3 overexpression vectors were suspended in PBS and injected into the right frontal node of nude mice. The inoculation position was 2 mm lateral and 2 mm posterior to the anterior fontanel. Tumor size was estimated from luciferase volume measurements and MRI. The mice were sacrificed when they exhibited disturbed activity or convulsion. The brain was then harvested and embedded in paraffin.

### Immunohistochemistry and immunofluorescent staining

Immunohistochemistry was performed as previously reported [8]. Briefly, sections were deparaffinized and boiled in Tris antigen-retrieval buffer. Then, sections were incubated with primary antibodies against YTHDF2 (Abcam, 1:200 dilution), METTL3 (Abcam, 1:200 dilution), and Ki-67 (Zsbio working solution, Beijing, China) overnight at 4 °C. Immunohistochemistry images were captured using an Axio Imager 2 (Zeiss).

The YTHDF2 immunoreactive scores were calculated according to the intensity score multiplied by the percentage score. The score of staining intensity was defined as follows: 0 for ‘no staining,’ 1 for weak staining, 2 for moderate staining, and 3 for strong staining. The percentage score was determined as follows: 0 for less than 5% cells positive, 1 for 5–25% cells positive, 2 for 26–50% cells positive, 3 for 51–75% cells positive, and 4 for greater than 75% cells positive.

Immunofluorescence staining was performed according to our previous report [26]. Cell cultures were incubated with primary antibodies against YTHDF2 (Abcam, 1:200 dilution) and NF-κB p65 (CST, 1:200 dilution) overnight at 4 °C. After washing, the cultures were incubated with Alexa Fluor 488- or 546-conjugated secondary antibodies (Alexa Fluor, Proteintech). Images were captured using a laser immunofluorescence microscope (Zeiss).

### Statistical analysis

One-way ANOVA was used to compare the expression levels of YTHDF2 or other targets among three or more groups, and *t* tests were used to compare the expression levels of YTHDF2 or other targets between two groups. The Kaplan–Meier method with a two-sided log-rank test was used to compare the overall survival of patients between different groups.

All statistical analyses were conducted using R v3.4.1 (https://www.r-project.org/) and Prism 7 (GraphPad Software Inc., La Jolla, CA). A value of *P* < 0.05 was considered statistically significant.

## Results

### YTHDF2 expression is elevated in gliomas with higher malignancy

**Fig. 1:**
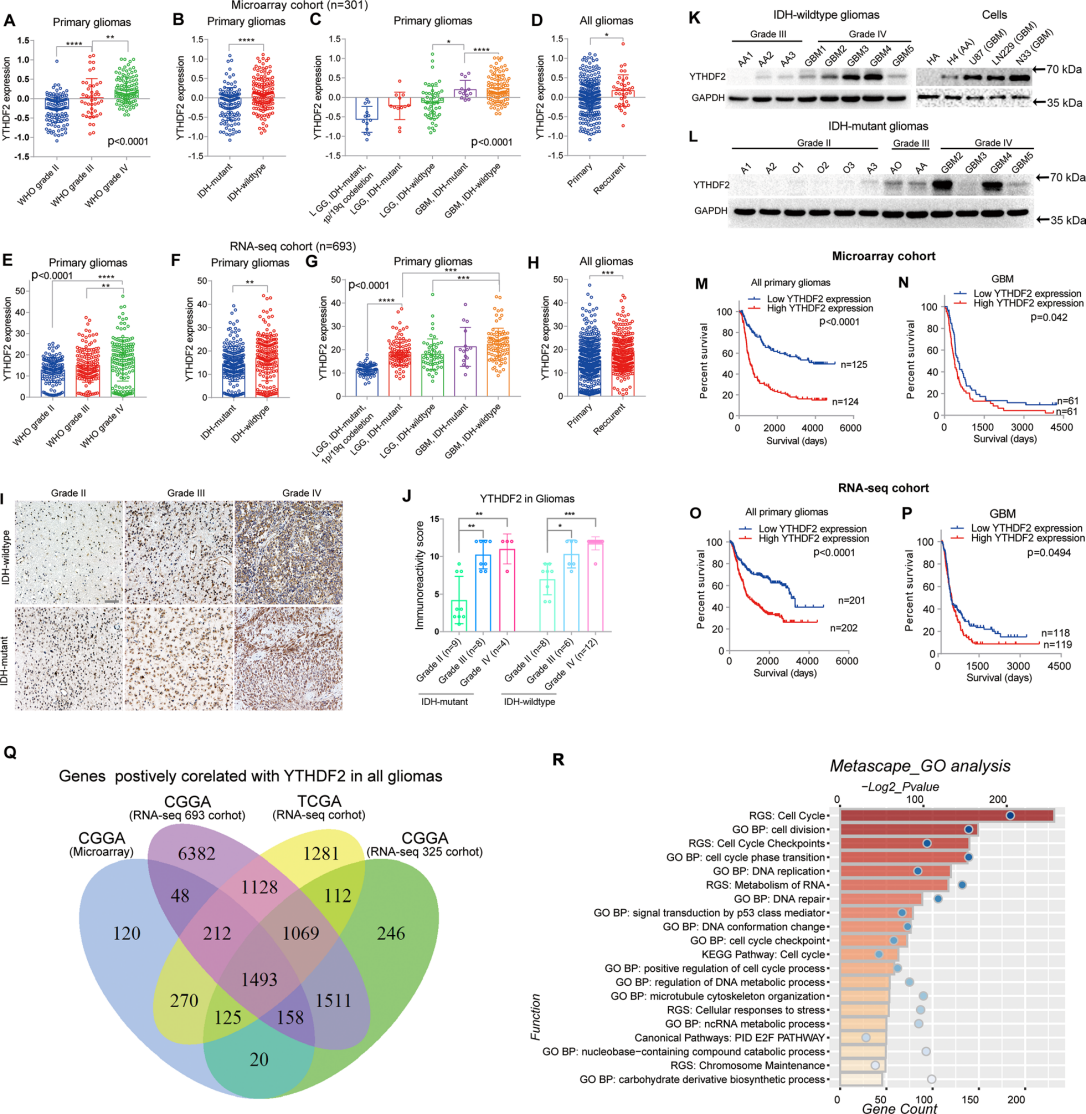
Expression and prognostic value of YTHDF2 in glioma. **A**–**C** The mRNA expression of YTHDF2 in primary gliomas with different WHO grade (**A**), IDH-mutant status (**B**), and classifications in CGGA microarray cohort. *P < 0.05; **P < 0.01; ****P < 0.0001. **(D)** The mRNA expression of YTHDF2 in primary and recurrent gliomas in CGGA microarray cohort. *P < 0.05. **E**–**H** The mRNA expression of YTHDF2 in gliomas stratified by different clinicopathological features in CGGA RNA-seq cohort (*n* = 693). **P < 0.01; ***P < 0.001; ****P < 0.0001. **I** Representative immunohistochemistry images of YTHDF2 protein in gliomas with different IDH status and histological grades. Bar = 50 µm. **J** The semi-quantitative for the immunohistochemistry results of YTHDF2 proteins. ***P < 0.001; ****P < 0.0001. **K** The protein expression of YTHDF2 in IDH-wildtype gliomas and in vitro cell models with different histological grades. **L** The protein expression of YTHDF2 in IDH-mutant gliomas with different histological grade. **M**–**P** Kaplan–Meier curves of gliomas and GBM from CGGA microarray cohort (**M**, **N**) and CGGA RNA-seq cohort (**O**, **P**) stratified by YTHDF2 expression. **Q** Venn diagram shows the overlap genes whose expression is positively correlated with YTHDF2 expression in primary gliomas form different datasets. **R** GO analysis terms of the 1493 genes whose expression are positively correlated with YTHDF2 expression in primary gliomas from four datasets

Our previous study indicated that YTHDF2 mRNA levels were significantly increased with increasing WHO glioma grade in both CGGA and TCGA datasets [9]. Here, we confirmed YTHDF2 expression in two extra CGGA cohorts (Fig. 1A–H). YTHDF2 expression was increased in primary gliomas with higher WHO grade (Fig. 1A) and in *IDH*-wild-type gliomas (Fig. 1B) in the Microarray cohort (*n* = 301) of CGGA. YTHDF2 mRNA levels were also increased in molecular subgroups of primary gliomas with higher malignancy (Fig. 1C) and in recurrent gliomas compared with primary cases (Fig. 1D). We also observed a similar distribution of YTHDF2 mRNA in another CGGA dataset (RNA-seq, *n* = 693) (Fig. 1E–H).

Next, we analyzed YTHDF2 protein levels in gliomas with different *IDH* status and WHO grades. Immunohistochemistry of 47 glioma cases revealed that the level of YTHDF2 protein was increased with increasing WHO grades in both *IDH*-wild-type and *IDH*-mutant gliomas (Fig. 1I, J). Furthermore, Western blotting with freshly collected protein samples from eight *IDH*-wildtype gliomas showed that YTHDF2 protein levels were elevated in WHO grade IV tumors compared with WHO grade III tumors (Fig. 1K). The YTHDF2 protein levels in glioblastoma cell lines (LN229 and U87 cells) and glioblastoma patient-derived N33 cells were also higher than those in anaplasia astrocytoma H4 cells and human astrocytes (HA) (Fig. 1K). Similar results were observed with clinical samples of twelve *IDH*-mutant gliomas (Fig. 1L). Based on these findings, we also studied the prognostic value of YTHDF2 expression in the CGGA Microarray and newly released RNA-seq datasets (Fig. 1M–P). The overall survival of primary glioma patients with higher YTHDF2 expression was poorer than that of patients with low YTHDF2 expression in both datasets. This is consistent with our previous findings in TCGA and another CGGA RNA-seq datasets [9]. In addition, in both datasets, the overall survival of GBM patients could also be stratified by YTHDF2 expression.

We and others have reported that m^6^A ‘writers,’ particularly METTL3, also play important roles in the tumorigenesis and malignant progression of glioma [7, 27–29]. Here, we also studied the expression of m^6^A ‘writers,’ including METTL3, METTL14, and WTAP, in these two additional datasets. Elevated METTL3 mRNA expression was observed in *IDH*-mutant gliomas in both cohorts, consistent with our recent report [27] (Additional file 1: Fig. S1A). The mRNA expression of METTL14 showed an increasing trend in *IDH*-wildtype GBM in the Microarray cohort, but there was no significant difference among different glioma subtypes in the RNA-seq cohort (Additional file 1: Fig. S1B). Remarkably, the expression of WTAP increased in gliomas with higher malignancy, such as higher WHO grade, *IDH*-wildtype, and molecular subgroups with poorer survival, in both tow datasets (Additional file 1: Fig. S1C). Next, we investigated the prognostic value of mRNA expression levels of these m^6^A ‘writers’ in gliomas. Interestingly, METTL3 showed opposite prognostic value in gliomas of the RNA-seq and Microarray cohorts (Additional file 1: Fig. S1D–G). This may be due to the different proportion of high-grade *IDH*-wildtype cases in these two cohorts. METTL14 did not stratify survival of gliomas in either RNA-seq or Microarray cohorts (Additional file 1: Fig. S1H–K). Consistent with the distribution of WTAP expression, primary glioma patients with higher WTAP expression had poorer overall survival in both datasets (Additional file 1: Figure S1L, M). However, unlike YTHDF2*,* the mRNA expression levels of WTAP had no prognostic value for GBM in either dataset (Additional file 1: Fig. S1N, O).

The above findings suggest that the elevated expression of YTHDF2 may play an oncogenic role in glioma. To explore potential effects of YTHDF2 expression on glioma functions, we identified the genes that positively correlated with YTHDF2 expression in all primary gliomas of four different datasets, including the CGGA RNA-seq (*n* = 325) and TCGA RNA-seq (*n* = 595) cohorts used in our previous study [9] and the extra two cohorts in this study (Fig. 1Q). We then annotated the functions of the 1493 overlapping genes using functional enrichment analysis with the Metascape tool [30]. These genes were enriched in malignancy-related biological processes, including cell cycle, metabolism of RNA, and cell cycle phase transition (Fig. 1R). We also performed similar analysis for GBM and showed that the overlapping genes were enriched in biological activities of cell cycle, cell division, cell cycle checkpoint, DNA replication, and metabolism of RNA (Additional file 1: Fig. S2A, B), while the genes that negatively correlated with YTHDF2 were mainly enriched in functions of synapse signaling and other neuronal related activities (Additional file 1: Fig. S2C).

Together, these findings indicated that YTHDF2 plays an oncogenic role in gliomas and its expression is positively associated with increased malignant phenotype of gliomas, and may influence the cell cycle of glioma cells through regulation of RNA metabolism.

### YTHDF2 affects the proliferation and migration of glioma cells

**Fig. 2:**
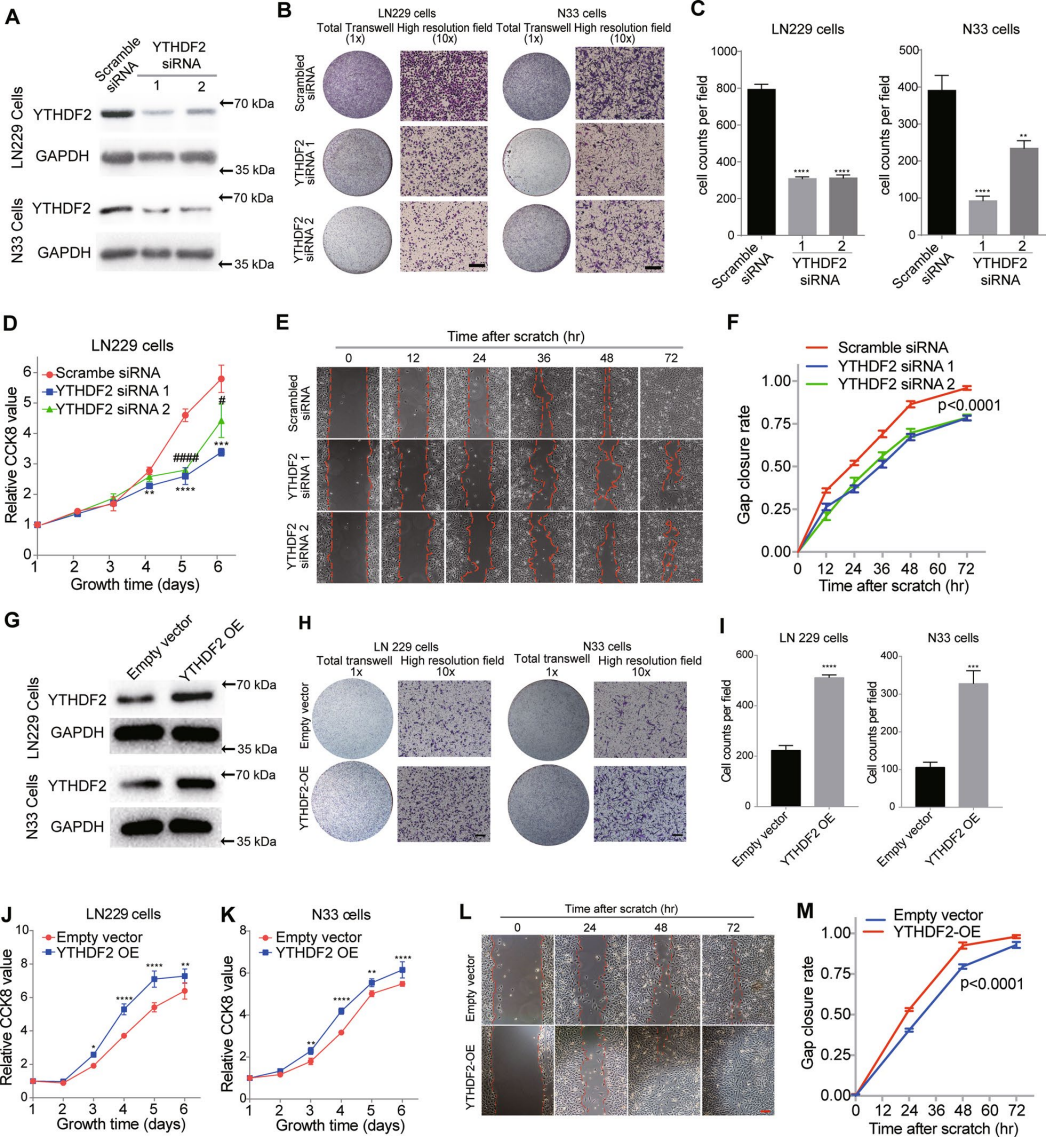
YTHDF2 could facilitate the malignant phenotype of glioma cells. **A** YTHDF2 protein expression in glioma cells with or without YTHDF2 siRNA. All of raw images have been shown in Additional file 3. **B**, **C** Transwell analysis of cells with or without YTHDF2 siRNA. **P < 0.01 and ***P < 0.001. Bar = 50 μm. **D** CCK-8 assay (proliferation assay) of cells with or without YTHDF2 siRNA. YTHDF2 siRNA 1 versus scrambled siRNA. **P < 0.01; ***P < 0.001; ****P < 0.0001; YTHDF2 siRNA2 versus scrambled siRNA. ^#^*P* < 0.05; ^####^*P* < 0.0001. **E** Scratch assay of LN229 cells with or without YTHDF2 siRNA. Bar = 50 μm. **F** Gap closure rate of cells with or without YTHDF2 siRNA. **G** YTHDF2 protein expression in glioma cells with or without YTHDF2 overexpression. **H**, **I** Transwell analysis of cells with or without YTHDF2 overexpression. ***P < 0.001 and ****P < 0.0001. Bar = 50 μm. **J**, **K** CCK-8 assay (proliferation assay) of cells with or without YTHDF2 overexpression. *P < 0.05; **P < 0.01; and ****P < 0.0001. **L** Scratch assay of LN229 cells with or without YTHDF2 overexpression. Bar = 50 μm. **M** Gap closure rate of cells with or without YTHDF2 overexpression

To study the influence of YTHDF2 expression levels on glioma cells, we successfully knocked down YTHDF2 expression in LN229 and N33 cells using specific siRNAs (Fig. 2A). We then performed Transwell migration assays and found that YTHDF2 knockdown significantly attenuated the migration of glioma cells (Fig. 2B, C). Next, CCK-8 assays indicated that YTHDF2 knockdown significantly attenuated the proliferation of LN229 cells (Fig. 2D). In addition, we validated the effects of YTHDF2 knockdown on the proliferation and migration of LN229 cells using a scratch-wound model. These results demonstrated that YTHDF2 knockdown significantly suppressed the gap closure rate of GBM cells (Fig. 2E, F and Additional file 1: Fig. S3A).

We also overexpressed YTHDF2 in LN299 and N33 cells through stable transfection of YTHDF2-overexpressing lentivirus (Fig. 2G). Transwell migration assays showed that YTHDF2 overexpression enhanced the migration of glioma cells (Fig. 2H, I). CCK-8 assays demonstrated that YTHDF2 overexpression significantly increased the proliferation of LN229 and N33 cells (Fig. 2J, K). Meanwhile, the scratch-wound model showed that YTHDF2 overexpression accelerated the gap closure of LN229 cells (Fig. 2L, M; Additional file 1: Fig. S3B). To further validate these findings, we knocked down YTHDF2 expression in U87 cells using YTHDF2 siRNA (Additional file 1: Fig.S4A). YTHDF2 knockdown significantly suppressed migration and proliferation of U87 cells (Additional file 1: Fig. S4B, C). In contrast, YTHDF2 overexpression significantly enhanced migration and proliferation of U87 cells (Additional file 1: Fig. S4D–F). Taken together, these findings demonstrated that elevated expression of YTHDF2 promotes glioma cell malignancy.

### Elevated YTHDF2 induces NF-κB activation via suppression of UBXN1 expression

**Fig. 3:**
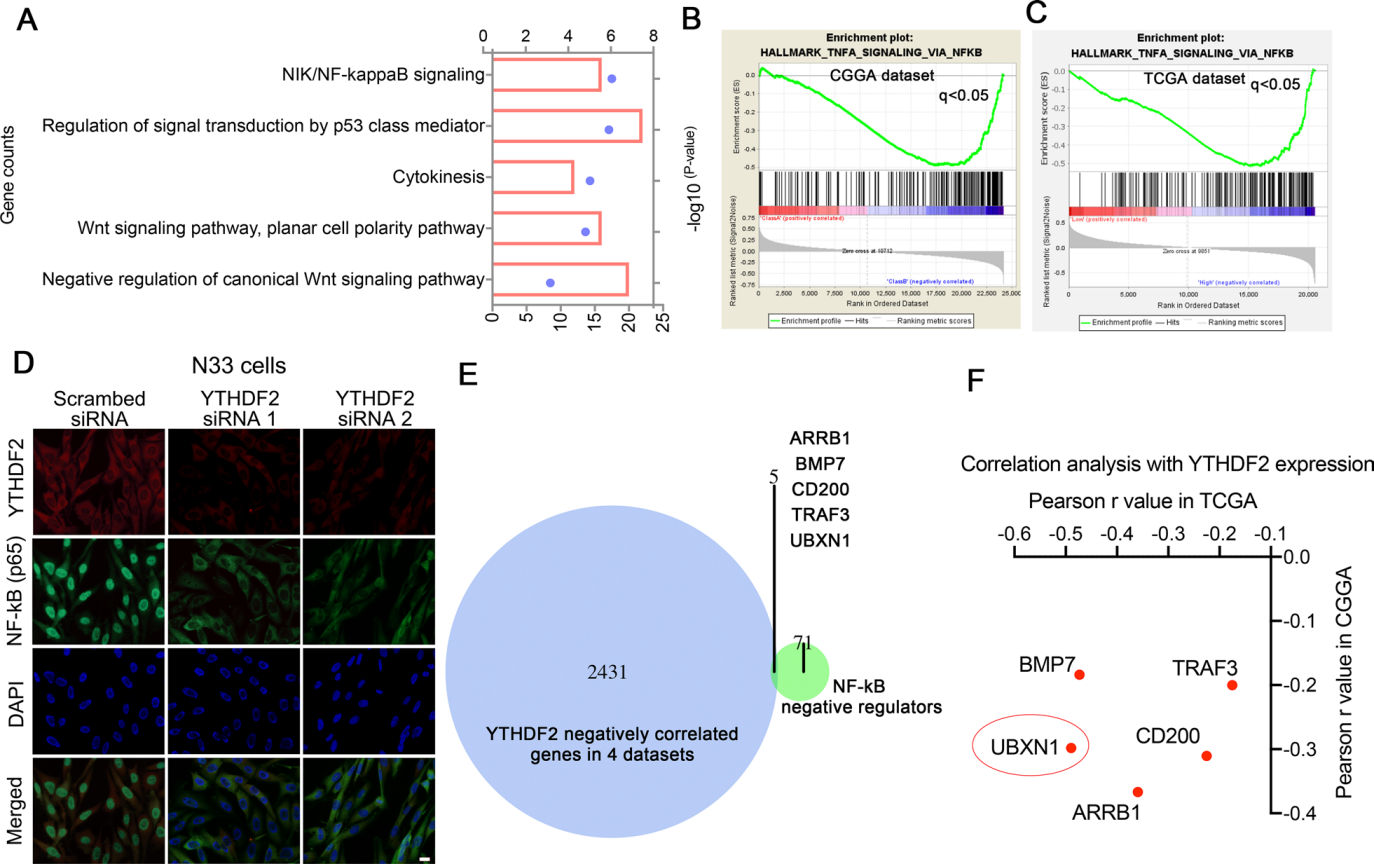
YTHDF2 may active NF-κB through suppressing its negative regulators. **A** KEGG pathways of the genes that positively correlated with YTHDF2 expression in gliomas from four datasets. The blue point shows the − log *P* value, and the red box shows the gene counts in each terms. **B**, **C** GSEA of hallmarks genes with increased expression in YTHDF2 high expression groups. **D** Immunostaining image showing NF-κB (p65) protein expression and localization in N33 cells with or without YTHDF2 siRNA. Bar = 10 μm. **E** Venn diagram shows the overlap genes whose expression is negatively correlated with YTHDF2 expression in primary gliomas form different datasets and negatively regulate NF-κB signaling. **F** Correlation of YTHDF2 expression and the 5 selected genes in gliomas from CGGA microarray and TCGA RNA-seq datasets

To further explore the potential pathways that underlying the oncogenic role of YTHDF2 in gliomas, we analyzed the enriched pathways of genes that positively associated with YTHDF2 expression in all four datasets. NF-κB signaling was the most significantly (with lowest *P* value) enriched pathway (Fig. 3A). GSEA showed that TNFα signaling via NF-κB was significantly enriched in cases with high YTHDF2 levels in both CGGA (Fig. 3B) and TCGA (Fig. 3C) datasets. Then, we observed the expression and distribution of NF-κB (p65) by immunostaining in N33 and LN229 cells with or without siRNA. The nuclear distribution of NF-κB (p65) was decreased in both N33 (Fig. 3D) and LN229 cells (Additional file 1: Fig. S5) treated with METTL3 siRNA.

Considering that the most well-characterized role of YTHDF2 is accelerating mRNA decay via recognition of m^6^A modification, we hypothesis that YTHDF2 may activate NF-κB via degrading RNA of genes which negatively regulate NF-κB. In the above study on the potential function of YTHDF2, we have identified 2437 genes that were significantly negatively associated with YTHDF2 expression in gliomas in all four different data sets (Additional file 1: Fig. S2C and Additional file 2: Table S3). Consistent with a recent study, the mRNAs of HIVEP2 and NRIH3 that have been shown to be YTHDF2 targets are also included in this list [19]. We also collected 76 genes that negatively regulate the NF-κB signaling pathway from the GO terminology and literatures (Additional file 2: Table S3). Further, we identified five targets that may be involved in the negative regulation of NF-κB by YTHDF2 via overlapping these two gene lists (Fig. 3E). Among the five genes, UBXN1 showed a relatively strong negative correlation with YTHDF2 in both CGGA microarray and TCGA RNA-seq datasets (Fig. 3F; Additional file 1: Fig. S6). Next, we focused on the relationship between YTHDF2 and UBXN1, which could negatively regulate activation of NF-κB via different ways [20, 31].

**Fig. 4:**
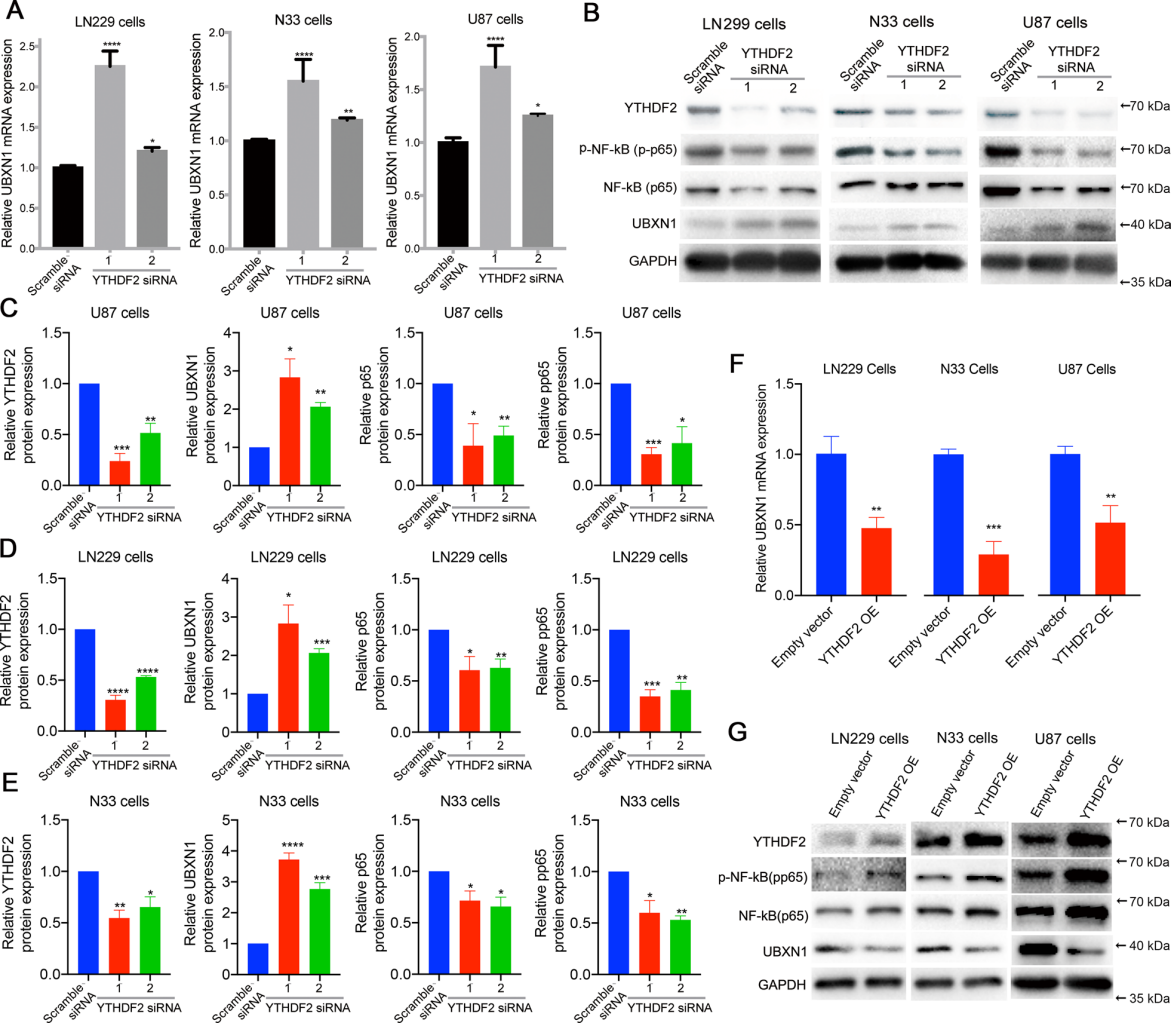
YTHDF2 can activate NF-κB through suppressing UBXN1 expression. **A** UBXN1 mRNA expression in cells with or without YTHDF2 siRNA. *P < 0.05; **P < 0.01; ****P < 0.0001. **B** YTHDF2, p65, pp65, and UBXN1 protein expression in cells with or without YTHDF2 siRNA. **C–E** The quantified data of protein expression levels in cells with or without YTHDF2 siRNA. *P < 0.05; **P < 0.01; ***P < 0.001. **F** UBXN1 mRNA expression in cells with or without YTHDF2 overexpression (OE). **P < 0.01; ***P < 0.001. **G** YTHDF2, p65, pp65, and UBXN1 protein expression in cells with or without YTHDF2 OE

We observed that YTHDF2 knockdown could significantly upregulate UBXN1 expression in LN299, N33, and U87 cells (Fig. 4A). Meanwhile, we observed upregulated UBXN1 expression together with decreased phosphorylated-NF-κB (pp65) in YTHDF2 siRNA-treated LN299, N33 and U87 cells (Fig. 4B–E). We also validated this result in LN229 cells through two independent YTHDF2 specific shRNA. The results showed that YTHDF2 shRNA could also significantly up-regulated the mRNA and protein expression of UBXN1, accompany with the suppression of NF-κB activation (Additional file 1: Fig. S7). Additionally, YTHDF2 overexpression significantly down-regulated *UBXN1* mRNA levels in LN299, N33 and U87 cells (Fig. 4F). Western blotting also showed that YTHDF2 overexpression down-regulated UBXN1 and up-regulated pp65 levels in these cells (Fig. 4G). These findings demonstrated that YTHDF2 upregulation can induce NF-κB activation via suppression of UBXN1 expression.

### YTHDF2 accelerates UBXN1 mRNA decay in gliomas via METTL3-mediated m^6^A modification

To explore whether there are m^6^A modification sites on UBXN1 mRNA that have the potential to be recognized by YTHDF2 in glioma cells, we performed MeRIP sequencing in GBM patient-derived N33 cells. Reads enriched in m^6^A antibody-precipitated RNA mapped to three regions on *UBXN1* mRNA, and the region around the 3′ end of *UBXN1* mRNA had the highest enrichment score (Additional file 1: Fig. S8A). Meanwhile, by checking the database of epitranscriptomic targets of readers, erasers, and writers, we also found that the m^6^A modification sites around the 3′ end of *UBXN1* mRNA have the potential to be recognized by YTHDF2 (Additional file 1: Fig. S8B and C).

**Fig. 5:**
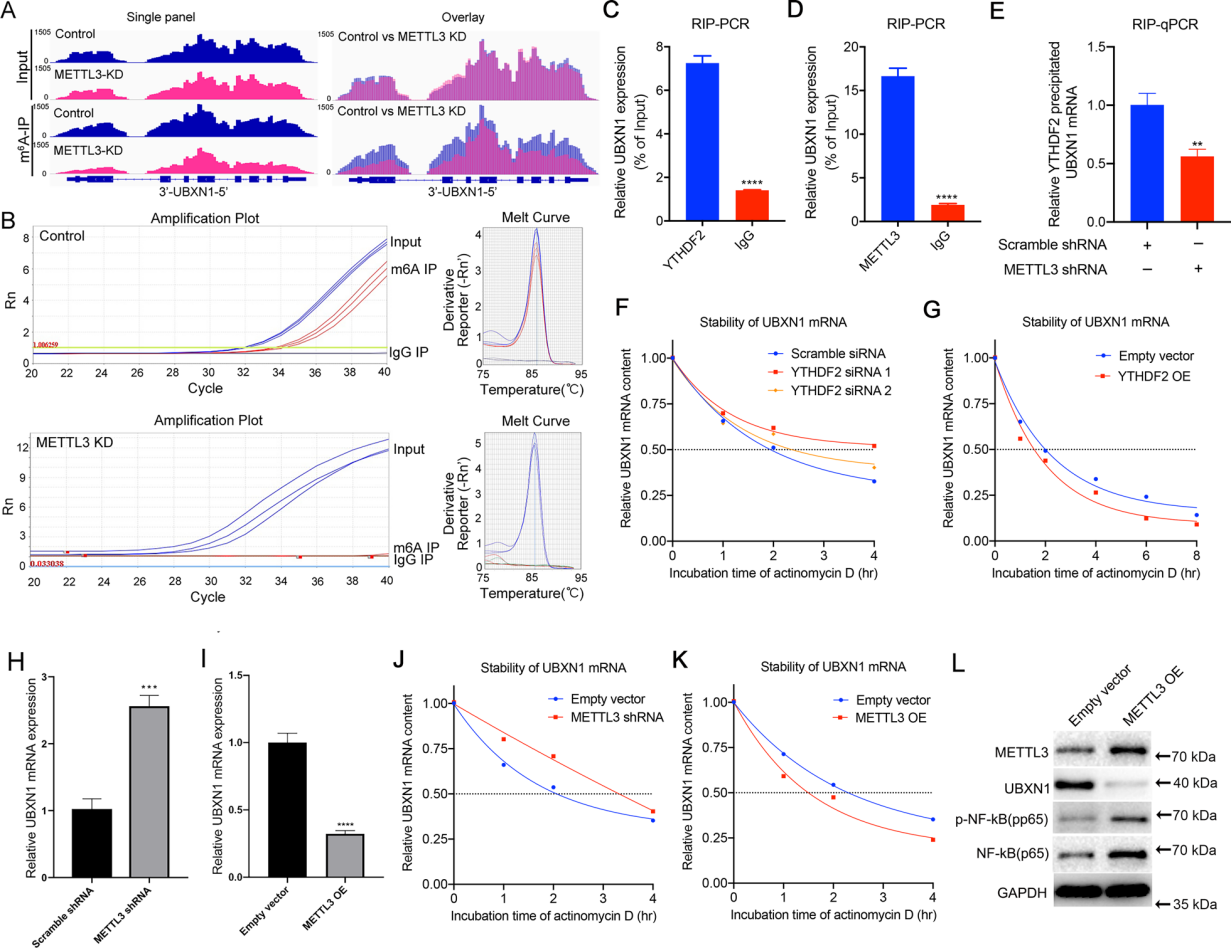
YTHDF2 can facilitate UBXN1 mRNA decay through recognizing m^6^A modification on UBXN1 mRNA that mediated by METTL3. **A** MeRIP-sequencing data of UBXN1 in U87 cells with or without shRNA-mediated METTL3 knockdown (METTL3 KD). **B** MeRIP-PCR data shows the relative quantity of UBXN1 mRNA immunoprecipitated by the m^6^A antibody (m^6^A-IP) and IgG in cells with or without METTL3 KD. **P < 0.01; ***P < 0.001; **P < 0.0001. **C**, **D** RIP-PCR showing the content of UBXN1 mRNA immunoprecipitated by METTL3 and YTHDF2 antibodies. IgG antibodies were used as negative control. ****P < 0.0001. **E** RIP-qPCR showing the content of UBNX1 immunoprecipitated by YTHDF2 antibodies in U87 cells with or without METTL3 shRNA. **F** The stability of UBXN1 mRNA in U87 cells with or without YTHDF2 siRNA. **G** The stability of UBXN1 mRNA in U87 cells with or without YTHDF2 overexpression (OE). **H** The expression levels of UBXN1 mRNA in U87 cells with or without METTL3 shRNA. **I** The expression levels of UBXN1 mRNA in U87 cells with or without METTL3 OE. **J** The stability of UBXN1 mRNA in U87 cells with or without METTL3 shRNA. **K** The stability of UBXN1 mRNA in U87 cells with or without METTL3 OE. **L** METTL3, UBXN1, pp65, and p65 protein expression in U87 cells with or without YTHDF2 OE

METTL3 is a widely recognized “writer of m^6^A, and its expression determines the level of m^6^A modification of RNAs in glioma [27]. To further validate the existence of m^6^A modification on *UBXN1* mRNA in gliomas, we performed MeRIP-sequencing of U87 cells with or without METTL3 knockdown. The results revealed that there were peaks mapping to *UBXN1* mRNA in m^6^A antibody-precipitated RNA, and reads of peaks mapping to *UBXN1* mRNA were also decreased in METTL3-knockdown cells (Fig. 5A). Next, MeRIP-PCR showed a significant increase in *UBXN1* mRNA levels in the m^6^A antibody-precipitated RNA compared with that of IgG, and *UBXN1* mRNA precipitated by the m^6^A antibody was significantly decreased in METTL3-knockdown U87 cells (Fig. 5B). Meanwhile, RIP-PCR experiments also showed that *UBXN1* mRNA levels were significantly increased in RNA precipitated by METTL3 and YTHDF2 antibodies compared with that precipitated by IgG in U87 cells (Fig. 5C, D). Additionally, the *UBXN1* mRNA precipitated by YTHDF2 antibody was also significantly deceased in METTL3-knockdown cells (Fig. 5E). Together, these data indicate that there are METTL3-mediated m^6^A modification sites on *UBXN1* mRNA that have the potential to be recognized by YTHDF2 in glioma cells.

Next, we studied the impact of YTHDF2 knockdown and overexpression on the stability of *UBXN1* mRNA. The stability of *UBXN1* mRNA was increased in U87 cells by YTHDF2 knockdown (Fig. 5F) and decreased in cells overexpressing YTHDF2 (Fig. 5G). Both the expression and stability of *UBXN1* mRNA were also decreased in LN229 cells overexpressing YTHDF2 (Additional file 1: Fig. S9A, B). Meanwhile, we found that *UBXN1* mRNA levels were increased in METTL3 knockdown U87 cells, but decreased in U87 cells overexpressing METTL3 (Fig. 5H, I, Additional file 1: Fig. S9C, D). Additionally, the stability of *UBXN1* mRNA was also increased in METTL3 knockdown U87 cells (Fig. 5J) and decreased in cells overexpressing METTL3 (Fig. 5K). Western blotting also showed that UBXN1 expression was decreased and NF-κB activation was increased in U87 cells overexpressing METTL3 (Fig. 5L). In addition, we observed that the YTHDF2 knockdown could reverse the decreased expression of UBXN1 in U87 cells overexpressing METTL3 (Additional file 1: Fig. S10). We also investigated the relationship between METTL3 and UBXN1 expression in CGGA RNA-seq (*n* = 693) and TCGA RNA-seq (*n* = 595) cohorts. METTL3 expression was significantly negatively correlated (*r* = −0.3560, *P* < 0.0001) with UBXN1 expression in CGGA cohort (Additional file 1: Fig. S11A), while METTL3 expression positively correlated (*r* = 0.1925, *P* < 0.0001) to UBXN1 expression in TCGA cohort (Additional file 1: Fig. S11B). Considering that the impact of m^6^A ‘writer’ METTL3 on the expression level of UBXN1 may be dependent on m^6^A ‘reader’ YTHDF2, this inconsistent result may be caused by the difference of the distribution of YTHDF2 expression in the two cohorts. Next, we analyzed the correlation between the sum of METTL3 and YTHDF2 expression and UBXN1 expression. The sum of METTL3 and YTHDF2 expression significantly negatively correlated with UBXN1 expression in both CGGA (*r* = −0.5376, *P* < 0.0001) and TCGA (*r* = −0.2086, *P* < 0.0001) datasets (Additional file 1: Fig. S11C, D).

Together, these findings showed that YTHDF2 can accelerate *UBXN1* mRNA decay in gliomas via METTL3-mediated m^6^A modification.

### UBXN1 overexpression reverses the influence of YTHDF2 on the malignant phenotype of tumors and NF-κB activation

**Fig. 6:**
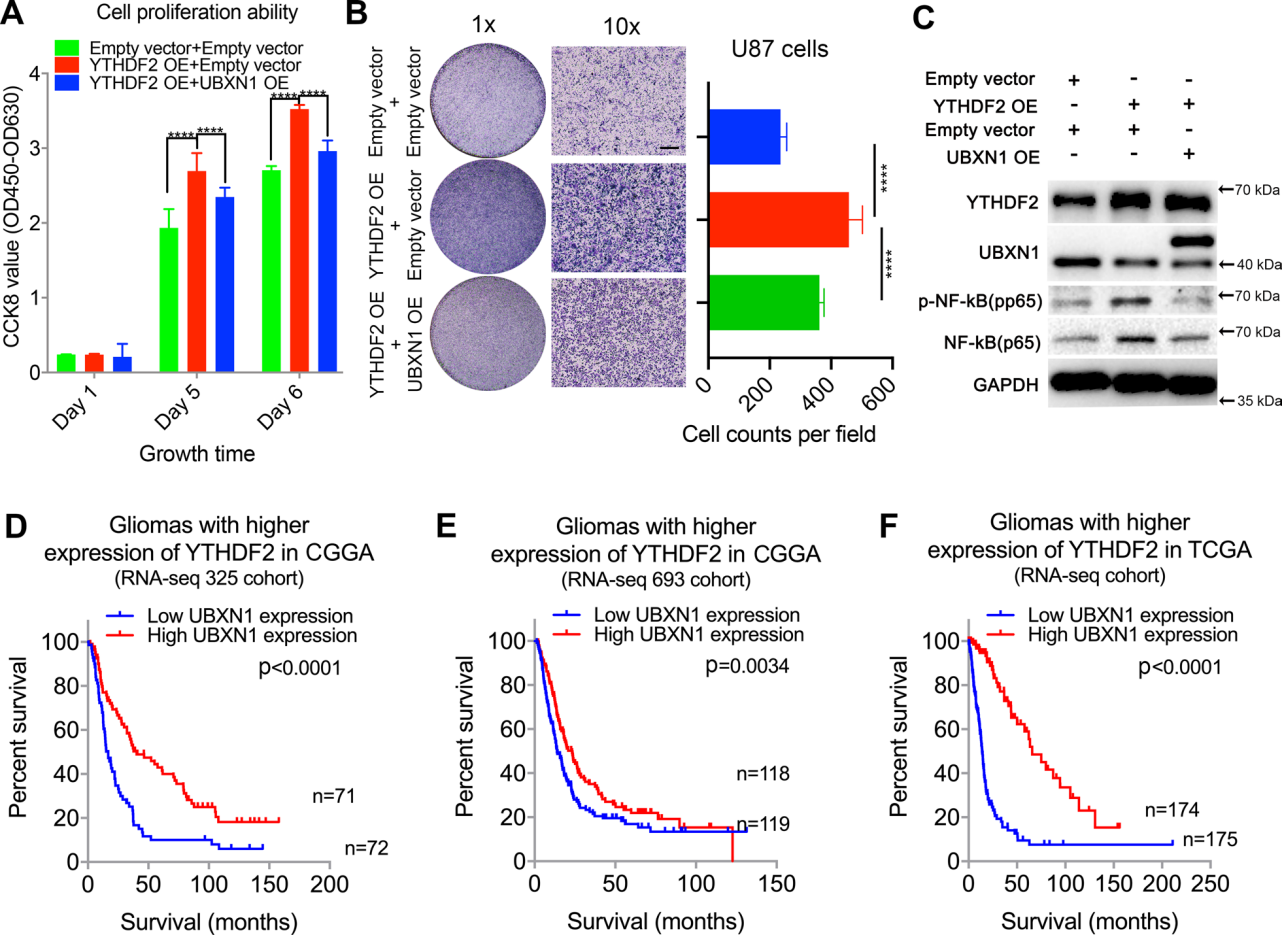
UBXN1 overexpression could attenuate malignant progression and NFKB activation that induced by YTHDF2 overexpression in glioma cells. **A** CCK-8 assay (proliferation assay) of U87 cells with or without YTHDF2 and UBXN1 OE ****P < 0.0001. **B** Transwell analysis of U87 cells with or without YTHDF2 and UBXN1 OE. ***P < 0.001. Bar = 50 μm. **C** YTHDF2, UBXN1, pp65, and p65 protein expression in U87 cells with or without YTHDF2 and UBXN1 OE. **D–F** Kaplan–Meier curves of gliomas with higher (larger than the median expression levels of respective cohorts) expression YTHDF2 stratified by UBXN1 expression in different datasets

To further validate the role of UBXN1 in YTHDF2 promotion of glioma progression and NF-κB activation, we overexpressed UBXN1 in cells overexpressing YTHDF2. CCK-8 assays indicated that UBXN1 overexpression significantly attenuated the proliferation of U87 cells overexpressing YTHDF2 (Fig. 6A). Meanwhile, transwell analysis showed the UBXN1 overexpression attenuated the increased migration of U87 cells caused by YTHDF2 overexpression (Fig. 6B). Western blotting also showed that UBXN1 overexpression suppressed NF-κB activation induced by YTHDF2 overexpression (Fig. 6C). These findings indicated that UBXN1 overexpression reversed the influence of YTHDF2 on the malignant phenotype of tumors and NF-κB activation. We also observed that *UBXN1* mRNA levels were significantly stratified with the prognosis of glioma cases with high YTHDF2 expression (higher than the median value) in several datasets (Fig. 6D–F).

### YTHDF2 and METTL3 promote tumor growth, poor survival, and NF-κB activation via suppression of UBXN1 expression in vivo

**Fig. 7:**
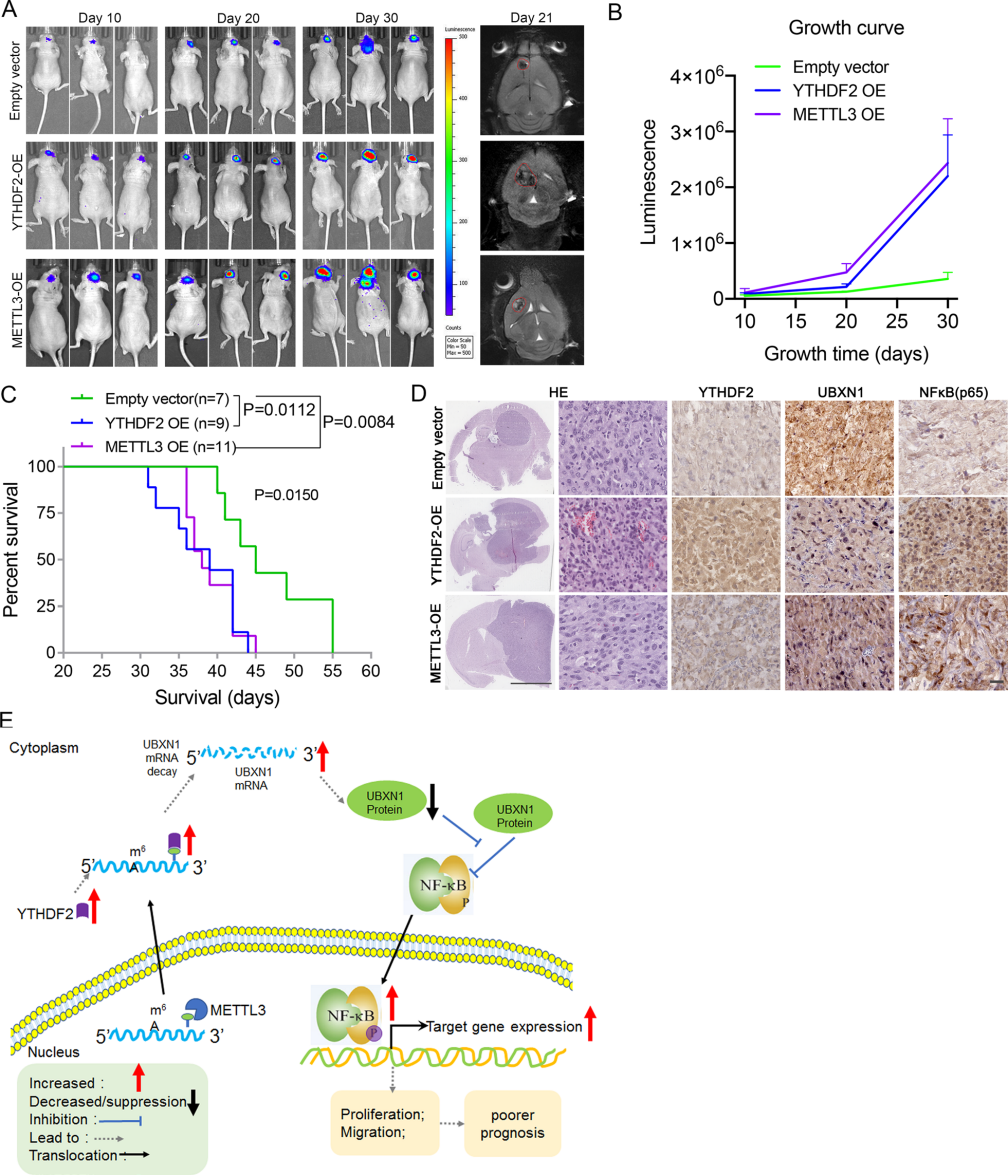
YTHDF2 and METTL3 promotes tumor growth and leads to a worse prognosis in an orthotopic xenograft model. **A** Representative tumor bioluminescence images of mice at 10, 20, and 30 days after tumor implantation in an orthotopic xenograft model generated by U87 cells transfected with an empty vector, YTHDF2 OE, and METTL3 OE vector. The representative magnetic resonance imaging of mice at 21 days after tumor implantation were also shown in the right panel. **B** The bioluminescence intensity of mice at 10, 20, and 30 days after tumor implantation. **C** Kaplan–Meier survival curve of nude mice with tumors. **D** Representative images of H&E and immunohistochemical staining for YTHDF2, UBXN1, and p65 in the tumors of nude mice. Bar = 3 mm in the left panel. Bar = 10 μm in the right panel. **E** The mechanistic scheme by which YTHDF2 facilitates UBXN1 mRNA decay via recognizing m^6^A modification that mediated by METTL3 to activate NF-κB and promote tumor malignancy

To investigate the effect of YTHDF2 on the malignant phenotype of glioma via m^6^A modification in vivo, we injected luciferase-labeled U87 cells expressing an empty vector, a YTHDF2 overexpression vector, or a METTL3 overexpression vector into the brains of nude mice. Tumor progression was monitored by bioluminescence imaging at days 10, 20, and 30. Tumors were also confirmed by nuclear MRI at day 21 after injection. Compared with xenografts derived from cells expressing the empty vector, the YTHDF2- and METTL3-overexpressing xenografts showed accelerated tumor progression (Fig. 7A, B). Survival analysis also showed that mice with YTHDF2- and METTL3-overexpressing xenografts had poorer survival outcomes (Fig. 7C). In addition, we performed immunohistochemistry to study the expression of UBXN1 and NF-κB (p65) levels in the xenografts. UBXN1 levels were decreased in YTHDF2- and METTL3-overexpressing xenografts and colocalized with increased levels of NF-κB (p65) (Fig. 7D).

## Discussion

RNA m^6^A modification is an emerging field in the study of tumorigenicity and therapy resistance of glioma [7, 11, 27, 32]. Our previous study also showed that the expression pattern of m^6^A ‘writers,’ ‘erasers,’ and ‘readers’ are highly associated with malignant progression and prognosis of glioma in both CGGA and TCGA cohorts [9]. However, the exact functions of m^6^A ‘writers,’ ‘erasers,’ and ‘readers’ are still largely elusive in gliomas, such as both oncogenic and tumor suppressive roles of m^6^A writer METTL3 have been reported in GBM-derived stem cells (GSCs) [28, 33, 34]. To further understand and potentially interfere the malignant progression of glioma from the perspective of RNA m^6^A modification, it is still required to carefully verify the expression, prognostic value, potential biological functions, and underlying mechanisms of m^6^A ‘writers,’ ‘erasers,’ and ‘readers’ in different cohorts of gliomas. Here, based on our previous work, we investigated the expression levels and prognostic values of m^6^A reader YTHDF2 in additional two large cohorts of CGGA. We also verified the protein expression of YTHDF2 in gliomas with different histological grade and *IDH*-mutant status. We showed that both the mRNA and protein levels of YTHDF2 are positively correlated with increased grade of glioma malignancy and poor prognosis. Based on this, we showed that the elevated expression of YTHDF2 can promote the proliferation and migration of differentiated GBM cells by promoting NF-κB activation both in vitro and in vivo. Mechanistically, YTHDF2 accelerated m^6^A-dependent degradation of *UBXN1* mRNA, which is a negative regulator of NF-κB activation.

Consistent with our previous report, a recent study also showed that YTHDF2 expression is increased in GBM and has prognostic value in pan-gliomas [9, 19]. However, *IDH*-mutant status can also dramatically impact the expression levels, prognostic values, and functions of m^6^A ‘writers,’ ‘erasers,’ and ‘readers’ in gliomas. *IDH* mutation can inhibit the activity of m^6^A ‘eraser’ FTO in leukemia and glioma cells by producing R-2-hydroxyglutarate [35, 36]. Our recent findings also demonstrate that METTL3 expression is significantly deceased in *IDH*-wildtype glioma, and METTL3 expression is positively associated with a higher malignant grade and poorer prognosis of *IDH*-wildtype gliomas but not *IDH*-mutant gliomas [27]. Thus, we comprehensively investigated that expression level of YTHDF2 in gliomas with different histological grade, *IDH*-mutant status, WHO 2016 classifications, and primary/recurrent status in this study. Unlike METTL3, which only increases with grade in *IDH*-wildtype gliomas, we demonstrated that YTHDF2 increased along with increasing WHO grades in both *IDH*-mutant and *IDH*-wildtype gliomas. Furthermore, we revealed that the expression of YTHDF2 significantly increased in recurrent gliomas compared with primary gliomas. We also showed that the expression of YTHDF2 has prognostic value in GBM, excluding the potential impact of histological grade on survival analysis in previous studies. These findings provide more detailed and reliable information to further understand the roles of YTHDF2 in gliomas.

The specific biological functions and their mechanisms of YTHDF2 in glioma have not been reported until recently, mainly focusing on the studies of GSCs [18, 19]. YTHDF2 can also promote invasive growth of GSCs by enhancing cholesterol dysregulation [19]. Similar to METTL3, YTHDF2 expression is also decreased in differentiated glioma cells compared with GSCs [19, 28]. There have been reports suggesting that YTHDF2 plays oncogenic role in cancer stem cells but suppresses the proliferation of differentiated tumor cells in liver cancer [37, 38]. Thus, in addition to GSCs, it is also necessary to study the role of YTHDF2 in differentiated glioma cells. Here, we observed that YTHDF2 expression was also increased in GBM cell lines and differentiated tumor cells derived from GBM patient compared to human astrocytes and grade III glioma cell lines. We further demonstrated that elevated YTHDF2 expression promoted the proliferation and migration of these cells, which was also consistent with bioinformatics analysis of glioma RNA-seq data from four different cohorts. These results suggest that YTHDF2 also plays an oncogenic role in differentiated glioma cells.

YTHDF2 has been shown to accelerate the degradation of more than 3000 RNA targets, most of which are mRNAs, but which also include non-coding RNAs [15]. In gliomas, elevated YTHDF2 expression could suppress the expression of LXRα and HIVEP2 via m^6^A-dependent mRNA clearance, which is required for GSC growth, invasion, and tumor formation in vivo [19]. Here, we demonstrated that YTHDF2 can enhance NF-κB activation by accelerating UBXN1 m^6^A-dependent mRNA decay. UBXN1, a UBX domain-containing protein, suppresses NF-κB activation by maintaining the expression of IκB [21, 31]. Additionally, we identified UBXN1 as a prognostic factor for better survival of glioma patients with high YTHDF2 expression. We also showed that UBXN1 expression can weaken the proliferation and migration of GBM cells promoted by YTHDF2 overexpression. LXRα regulates cholesterol homeostasis through regulating uptake and efflux of cholesterol, and the intracellular cholesterol is essential for glioma proliferation and invasion [39, 40]. HIVEP2 is a transcription factor whose downstream target, SSTR2, inhibits cancer cell proliferation [41]. Collectively, the above information showed that restraining UBXN1, LXRα and HIVEP2 expressions may have a synergistic effect in promoting tumorigenesis, and YTHDF2 overexpression could promote the tumorigenesis of glioma via accelerating the decay of LXRα, HIVEP2 and UBXN1 mRNA simultaneously.

Recently, downregulation of UBXN1 expression was also reported to be involved in the mechanism by which EGFR promotes NF-κB activation and the mechanism through which the long non-coding RNA, PRADX, activates NF-κB [20, 21]. Meanwhile, EGFR-YTHDF2 coupling can stabilize the YTHDF2 protein, which can promote GBM tumorigenesis by enhancing degradation of LXRA and HIVEP2 mRNA [19]. Furthermore, HIVEPS is a transcription factor that regulates MYC, NF-κB, and TGF-β signaling [42]. Interestingly, YTHDF2 has also been shown to increase the stability of MYC in GSCs [18]. Together, these information again suggest that a more complex network may be underlying the role of YTHDF2 overexpression in promoting tumorigenesis via targeting three genes of LXRα, HIVEP2 and UBXN1 simultaneously, which still warrants further investigation.

Activated NF-κB can mediate the activation of STAT3, CEBPB, and TAZ by upregulating the expression of CD44, vimentin, and N-cadherin, thereby promoting the invasion, angiogenesis, and therapeutic resistance of glioma [3, 43–45]. The m^6^A writer, METTL3, plays a key role in the maintenance of GBM stem cell characteristics by regulating m^6^A enrichment in *ADAM19*, *SOX2*, and *SRSF* genes [28]. Recently, we reported that upregulation of METTL3 could activate NF-κB via regulating MALAT1 [27]. Here, our findings show that METTL3 is also responsible for m^6^A enrichment in *UBXN1* mRNA, and is therefore involved in modulating NF-κB activation. Overall, these findings indicate that m^6^A has the potential to modulate NF-κB in multiple ways, which should be clarified by further studies.

## Conclusions

In conclusion, our findings demonstrate that YTHDF2 expression in diffuse gliomas increases with greater histological or molecular features of malignancy. Elevated YTHDF2 promotes malignancy of gliomas in both in vitro and in vivo models. Mechanistically, YTHDF2 accelerates *UBXN1* mRNA decay in gliomas by recognizing the m^6^A modification mediated by METTL3. This, in turn, enhances NF-κB activation (Fig. 7E). Taken together, our findings confirmed the oncogenic role of YTHDF2 in diffuse gliomas and revealed a novel regulatory mechanism of UBXN1 and NF-κB from the perspective of m^6^A modification.

## Supplementary Information

**Additional file 1.** The primers and supplementary figures for this manuscript.

**Additional file 2.** Identified genes that were negatively correlated with YTHDF2 expression.

**Additional file 3.** The raw gel figures used in this manuscript.

## Data Availability

The datasets used and/or analyzed during the current study are available from the CGGA official website (http://www.cgga.org.cn/) and the TCGA database (https://tcga-data.nci.nih.gov/tcga/tcgaDownload.jsp), and other data could be available from the corresponding author on reasonable request.

## Abbreviations

m^6^A: *N*6,2′-*O*-Dimethyladenosine
YTHDF2: YTH N6-methyladenosine RNA binding protein 2
METTL3: Methyltransferase-like 3
UBXN1: UBX domain protein 1
RIP: RNA immunoprecipitation
MeRIP: Methylated RIP
IDH: Isocitrate dehydrogenase [NADP(+)]
GBM: Glioblastoma
OS: Overall survival
CGGA: Chinese Glioma Genome Atlas
TCGA: The Cancer Genome Atlas
NF-κB: Nuclear factor kappa B
HA: Human astrocytes
