# Supplementary material for: YTHDF2 facilitates UBXN1 mRNA decay by recognizing METTL3-mediated m6A modification to activate NF-κB and promote the malignant progression of glioma
Source: J Hematol Oncol. 2021 Jul 10;14:109. doi: 10.1186/s13045-021-01124-z (PMC8272379; doi:10.1186/s13045-021-01124-z)
Supplement: Supplementary file 1 — Additional file 1. The primers and supplementary figures for this manuscript. [file 13045_2021_1124_MOESM1_ESM.pdf]

Supplementary materials

Table S1. The primers used in this study

Targets	Sequences
YTHDF2 F	CCCTCACAGGCTTTGGTTCA
YTHDF2 R	GCTGTGTCTGTTGCCCTACT
UBXN1 F	GGACAAAGCAGAGAGAGCCA
UBXN1 R	GACACTGGTCATACTCCCGC
METTL3 F	TTTTCCGGTTAGCCTTCGGG
METTL3 R	GATAGAGCTCCACGTGTCCG

Table S2. The siRNAs used in this study

Targets	Sequences
YTHDF2 siRNA 1	GCCCAAUAAUGCAUAUACUtt
YTHDF2 siRNA 2	GCGGGUCCAUUACUAGUAAtt

Figure S1 The expression and prognostic value of m^{6}A writers in glioma

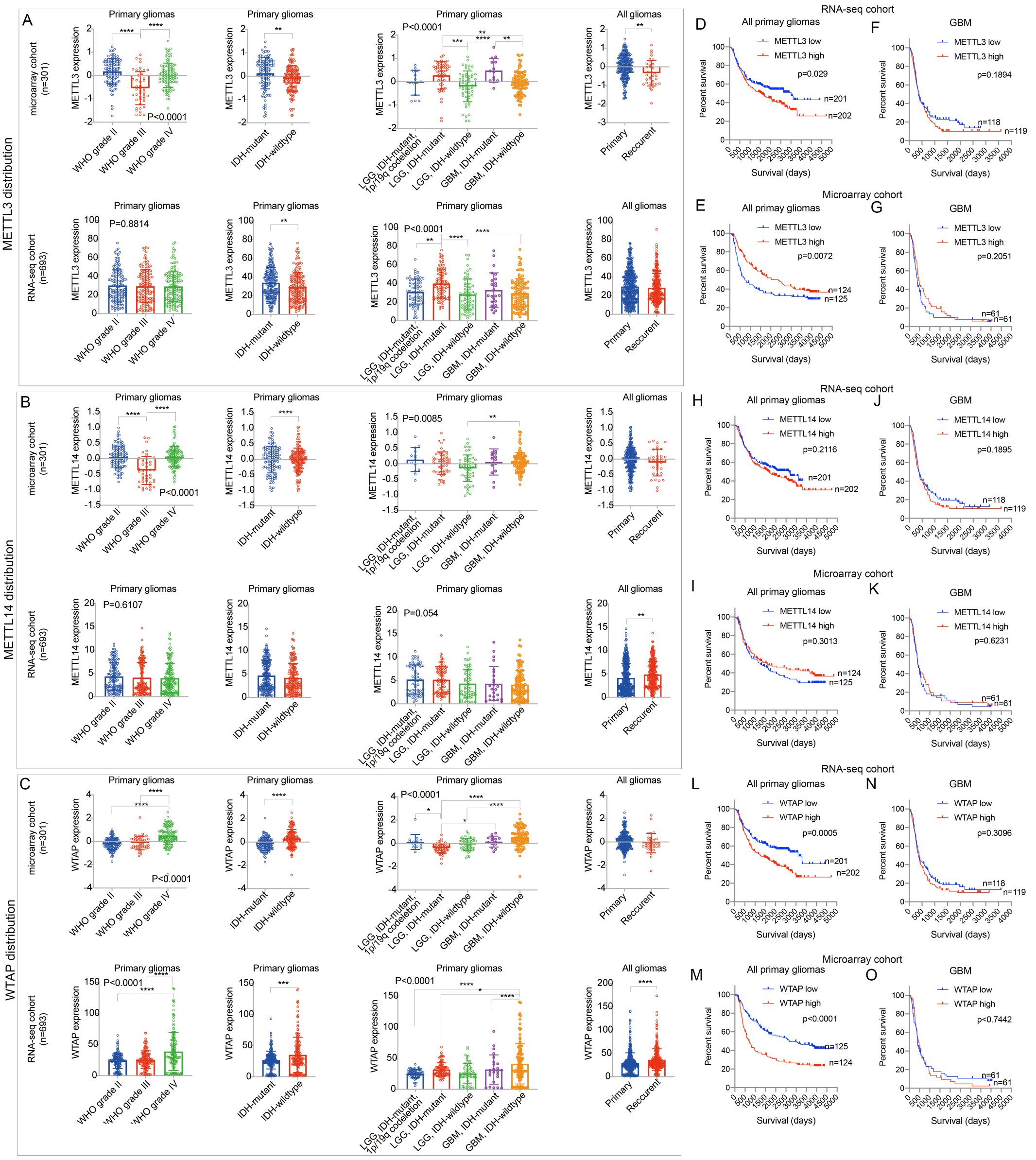

(A) The mRNA expression of METTL3 in gliomas with different clinical and pathological features in CGGA microarray cohort and a newly released CGGA RNA-seq cohort (n=693). **p<0.01, and ****p<0.0001. **(B)** The mRNA expression of METTL14 in gliomas with different clinical and pathological features in CGGA microarray cohort and a newly released CGGA RNA-seq cohort (n=693). **p<0.01, and ****p<0.0001. **(C)** The mRNA expression of WTAP in gliomas with different clinical and pathological features in CGGA microarray cohort and a newly released CGGA RNA-seq cohort (n=693). *p<0.05, **p<0.01, and ****p<0.0001. **(D-G)** Kaplan–Meier curves of gliomas and GBM from CGGA microarray cohort and CGGA RNA-seq cohort stratified by METTL3 expression. **(H-K)** Kaplan–Meier curves of gliomas and GBM from CGGA microarray cohort and CGGA RNA-seq cohort stratified by METTL14 expression. **(L-O)** Kaplan–Meier curves of gliomas and GBM from CGGA microarray cohort and CGGA RNA-seq cohort stratified by WTAP expression.

Figure S2 Functions of genes that positively correlated to YTHDF2 expression in glioma

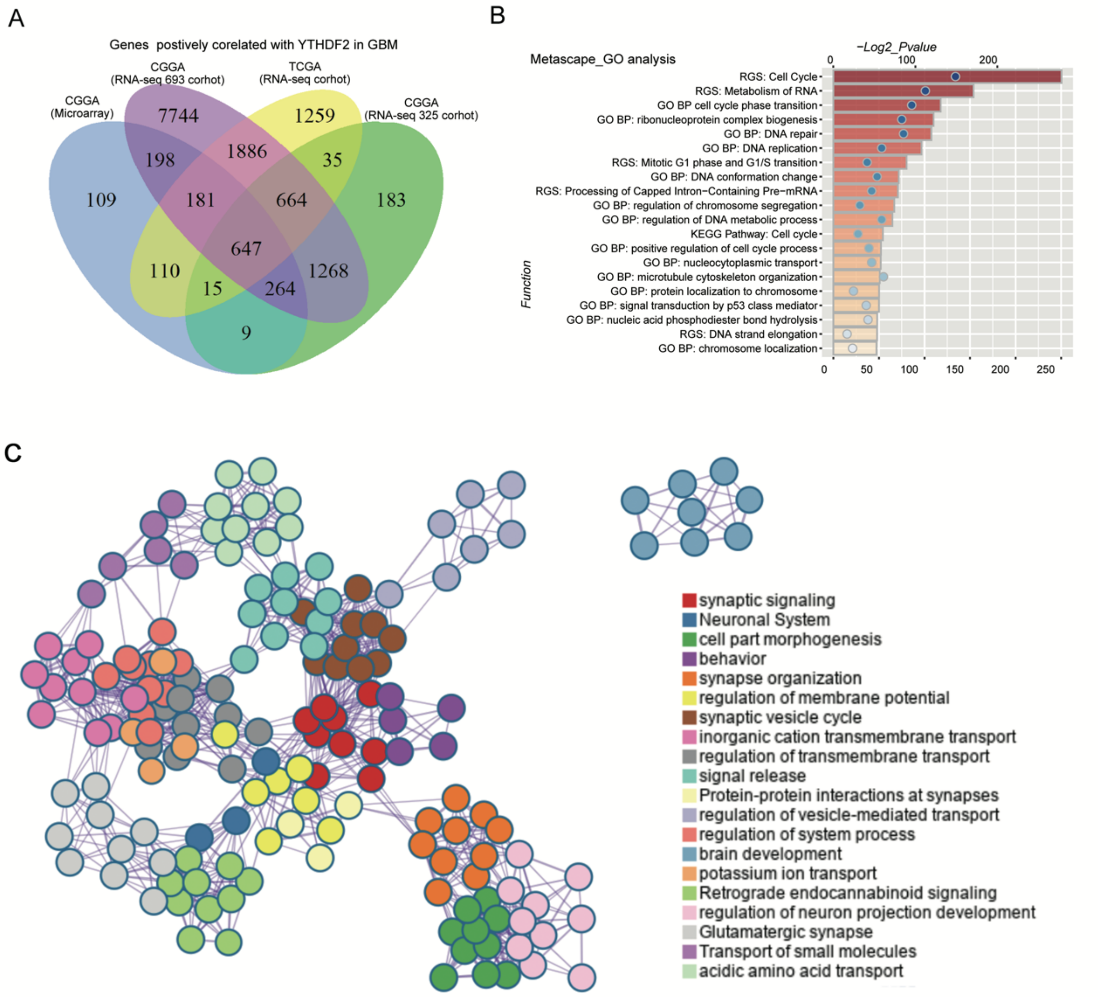

(A) Venn diagram shows the overlap genes whose expression is positively correlated with YTHDF2 expression in GBM form different datasets. **(B)** GO analysis terms of the 647 genes whose expression are positively correlated with YTHDF2 expression in GBM from four datasets. **(C)** functional network of genes whose expression are negatively correlated with YTHDF2 expression in all gliomas from four datasets.

Figure S3. Gap distances at different time after scratch

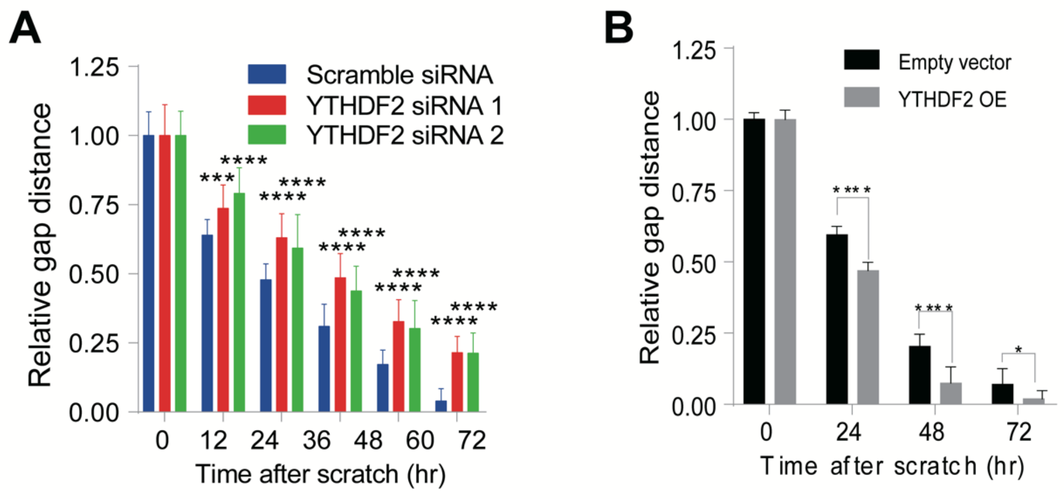

(A) The gap distances at different time after scratch in cells with our without YTHDF2 siRNA. ***p<0.001 and ****p<0.0001. **(B)** The gap distances at different time after scratch in cells with our without YTHDF2 OE. *p<0.05 and ****p<0.0001.

Figure S4. YTHDF2 could facilitate the malignant phenotype of U87 cells

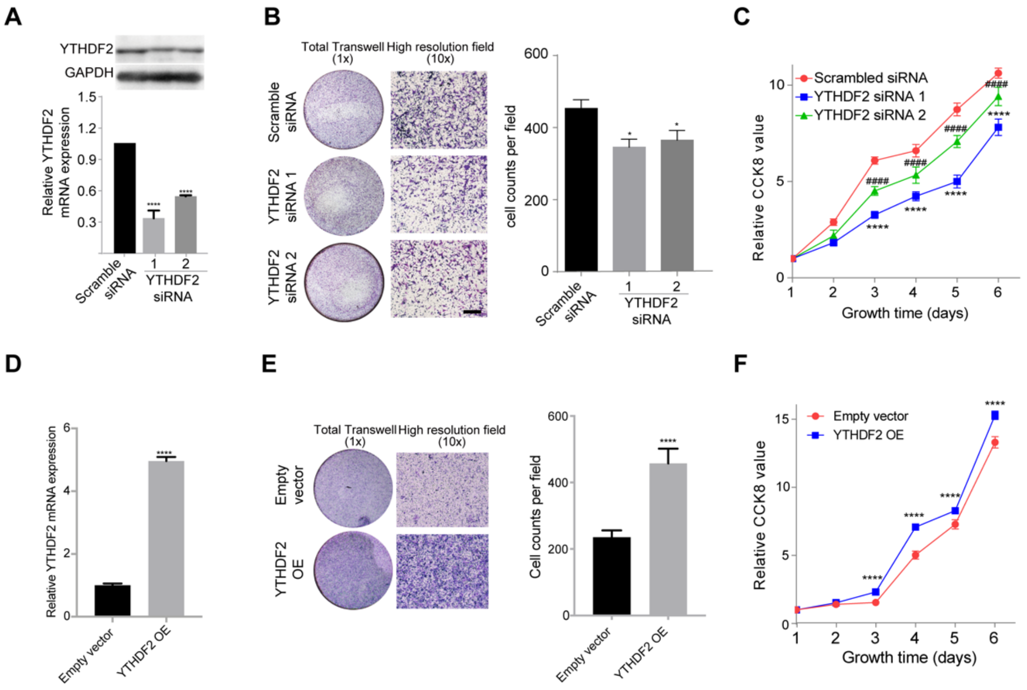

(A) YTHDF2 mRNA and protein expression in U87 cells with or without YTHDF2 siRNA. ****p<0.0001. **(B)** Transwell analysis of U87 cells with or without YTHDF2 siRNA. **p<0.01, ***p<0.001. Bar=50 μm. **(C)** CCK-8 assay (proliferation assay) of U87 cells with or without YTHDF2 siRNA. YTHDF2 siRNA 1 versus scrambled siRNA. ****p<0.0001; YTHDF2 siRNA 2 versus scrambled siRNA. ####p<0.0001. **(D)** YTHDF2 mRNA and protein expression in U87 cells with or without YTHDF2 overexpression. ****p<0.0001. **(E)** Transwell analysis of U87 cells with or without YTHDF2 overexpression. ****p<0.0001. Bar=50 μm. **(F)** CCK-8 assay (proliferation assay) of cells with or without YTHDF2 OE. ****p<0.0001.

Figure S5. NF-κB (p65) distribution in LN229 cells

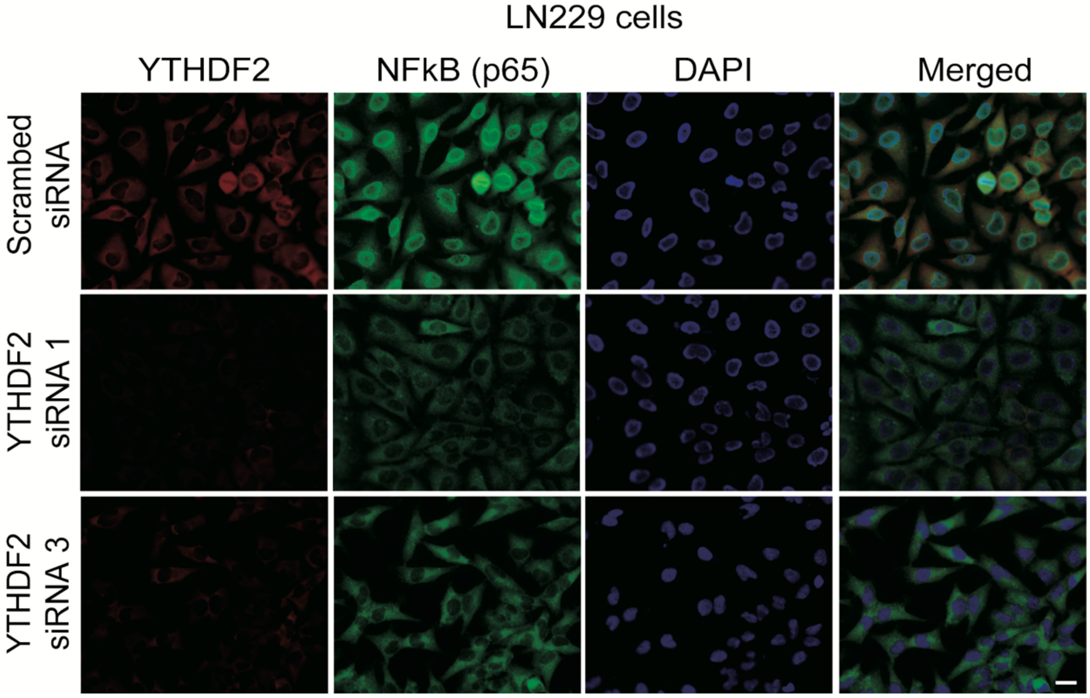

Immunostaining image showing NF-κB (p65) protein expression and localization in LN229 cells with or without YTHDF2 siRNA. Bar=10 μm.

Figure S6. Correlation of YTHDF2 expression and the 5 target genes

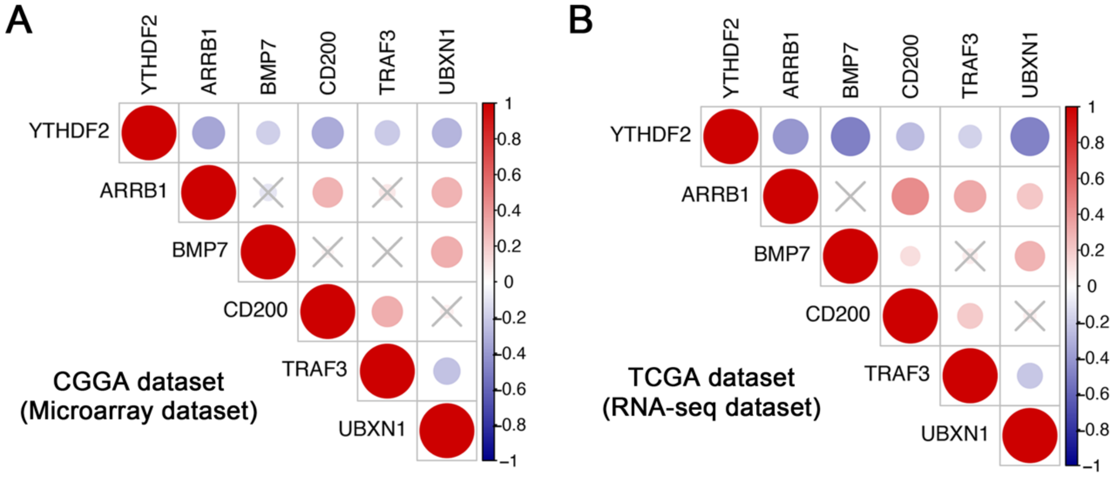

Correlation of YTHDF2 expression and the 5 selected genes in gliomas from CGGA microarray (A) and TCGA RNA-seq (B) datasets.

Figure S7. YTHDF2 shRNA could up-regulate UBXN1 mRNA and protein expression

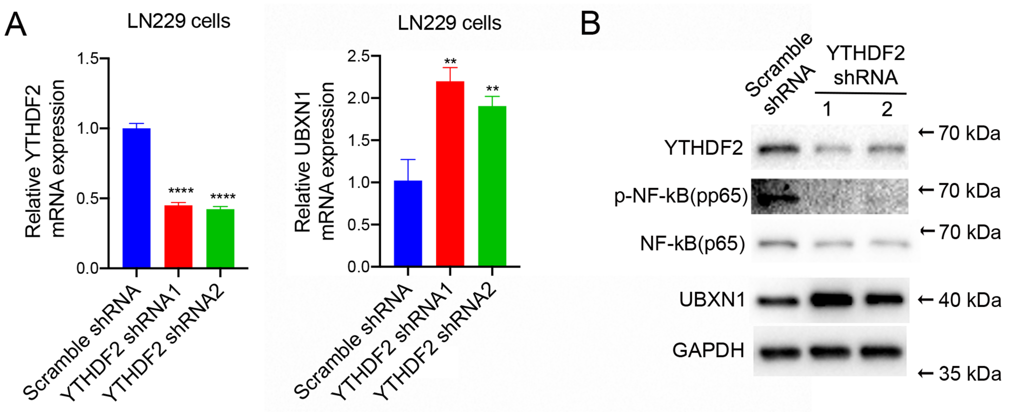

(A) YTHDF2 and UBXN1 mRNA expressions in LN229 cells with or without YTHDF2 shRNA. (B) YTHDF2, pp-65, p-65, UBXN1, and GAPDH protein expression in cells with or without YTHDF2 shRNA.

Figure S8. The m6A modification of UBXN1 mRNA in N33 cells.

A MeRIP-sequencing results in GBM patient derived N33 cells

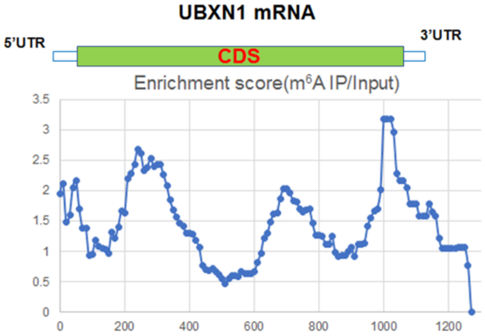

Peak	Peak range		
	Start	End	Length(bp)
1	191	480	290
2	661	890	230
3	961	1120	160

B The Epitranscriptomic Target of Reader, Eraser and Writer (TREW) database (https://zhenwei10.shinyapps.io/TREW/)

Regulator	Target_Gene	Regulator_Type	Mark	Species	Cellline	Technique	Positive_#	Reliability
Fto	Ubxn1	eraser	m6A	Mus musculus	3T3L1	MeRIP	1	100%
Fto	UBXN1	eraser	m6A	Mus musculus	3T3L1	MeRIP	1	100%
METTL3	UBXN1	writer	m6A	Mus musculus	MouseESC	MeRIP	1	100%
METTL3	Ubxn1	writer	m6A	Mus musculus	MouseESC	MeRIP	1	100%
YTHDF2	Ubxn1	reader	m6A	Mus musculus	MEF	MeRIP	1	100%
YTHDF2	UBXN1	reader	m6A	Mus musculus	MEF	MeRIP	1	100%

UBXN1 sequencing analysis

	Log2_RPKM	Modification	Diff_Pvalue	Diff_log2FoldChange	Start_position	Length (bp)
YTHDF2 WT	3.299	m6A	3.98+e5	5.81	988	5/52/117/4
YTHDF2 knockdown	2.885	m6A				

C The potential m^{6}A modification site on UBXN1 mRNA that may bind with YTHDF2

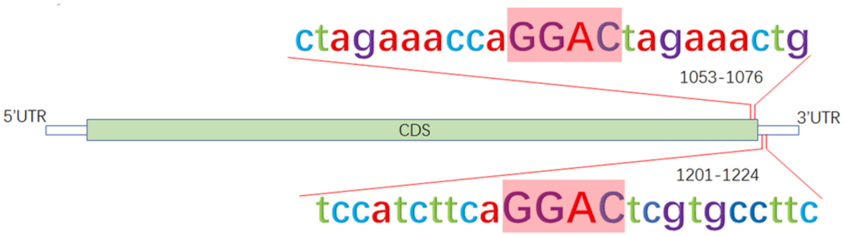

(A) MeRIP-sequencing data of UBXN1 mRNA represent potential modification peaks in N33 cells. **(B)** Analyzing the potential m^{6}A modification sites of UBXN1 mRNA that could be recognized by YTHDF2 in TREW database. **(C)** Potential m^{6}A modification sites of UBXN1 mRNA in 3' UTR region that may bind with YTHDF2.

Figure S9. YTHDF2 could facilitate UBXN1 mRNA decay in LN229 cells.

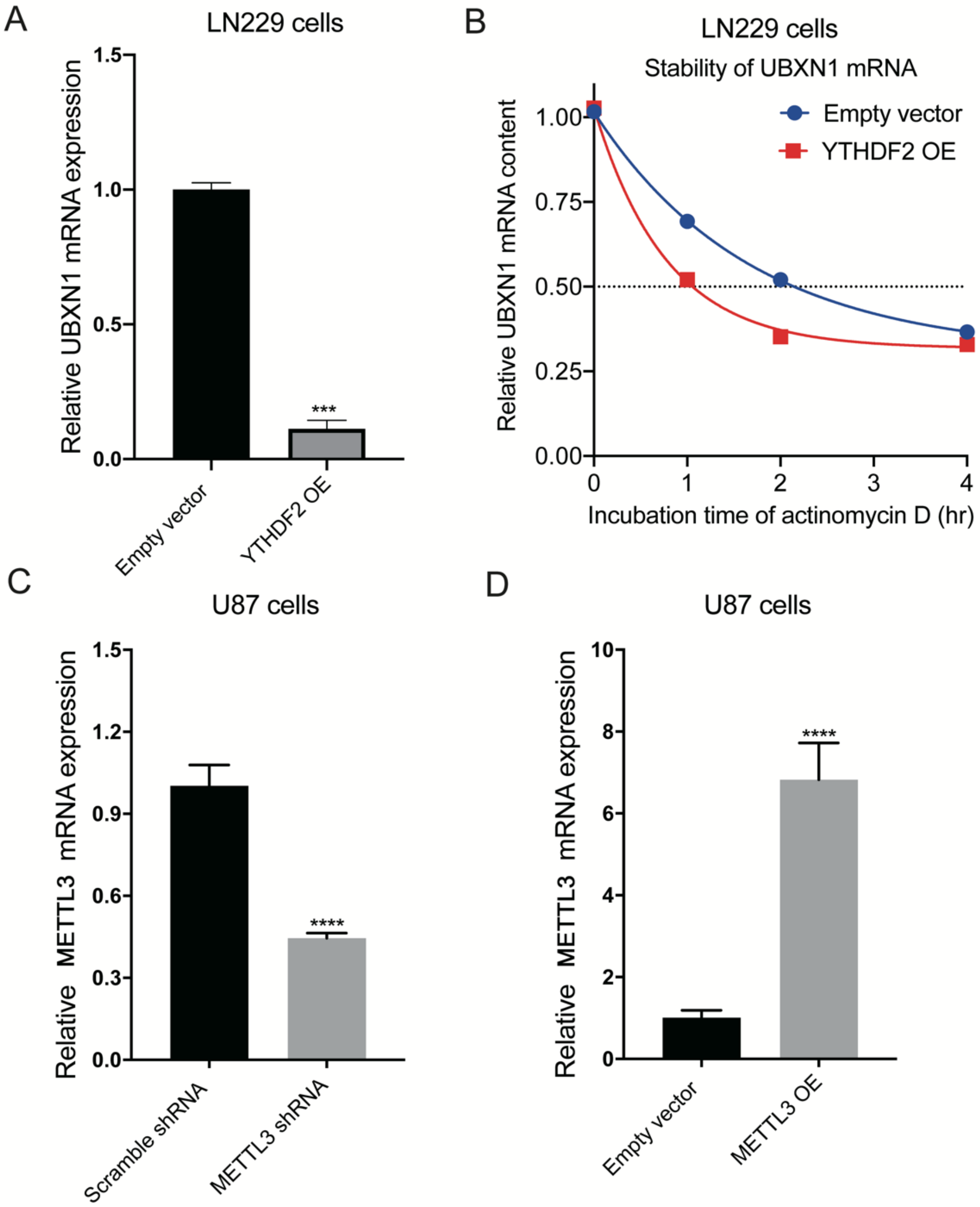

(A) The UBXN1 mRNA expression in LN229 cells with or without YTHDF2 OE. ***$p<0.001$. **(B)** The stability of UBXN1 mRNA in LN229 cells with or without YTHDF2 OE. **(C-D)** The METTL3 mRNA expression in U87 cells with or without METTL3 shRNA and with or without METTL3 OE. ****$p<0.0001$.

Figure S10. YTHDF2 could reverse the down-regulation of UBXN1 in METTL3 overexpressed cells.

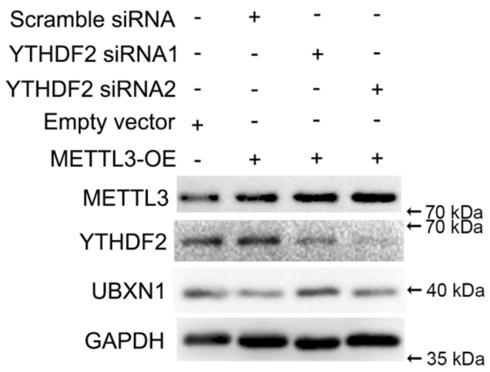

YTHDF2, UBXN1, METTL3 protein expression in U87 cells with or without METTL3 overexpression or YTHDF2 siRNA.

Figure S11. The correlation between METTL3 and UBXN1 expression in gliomas

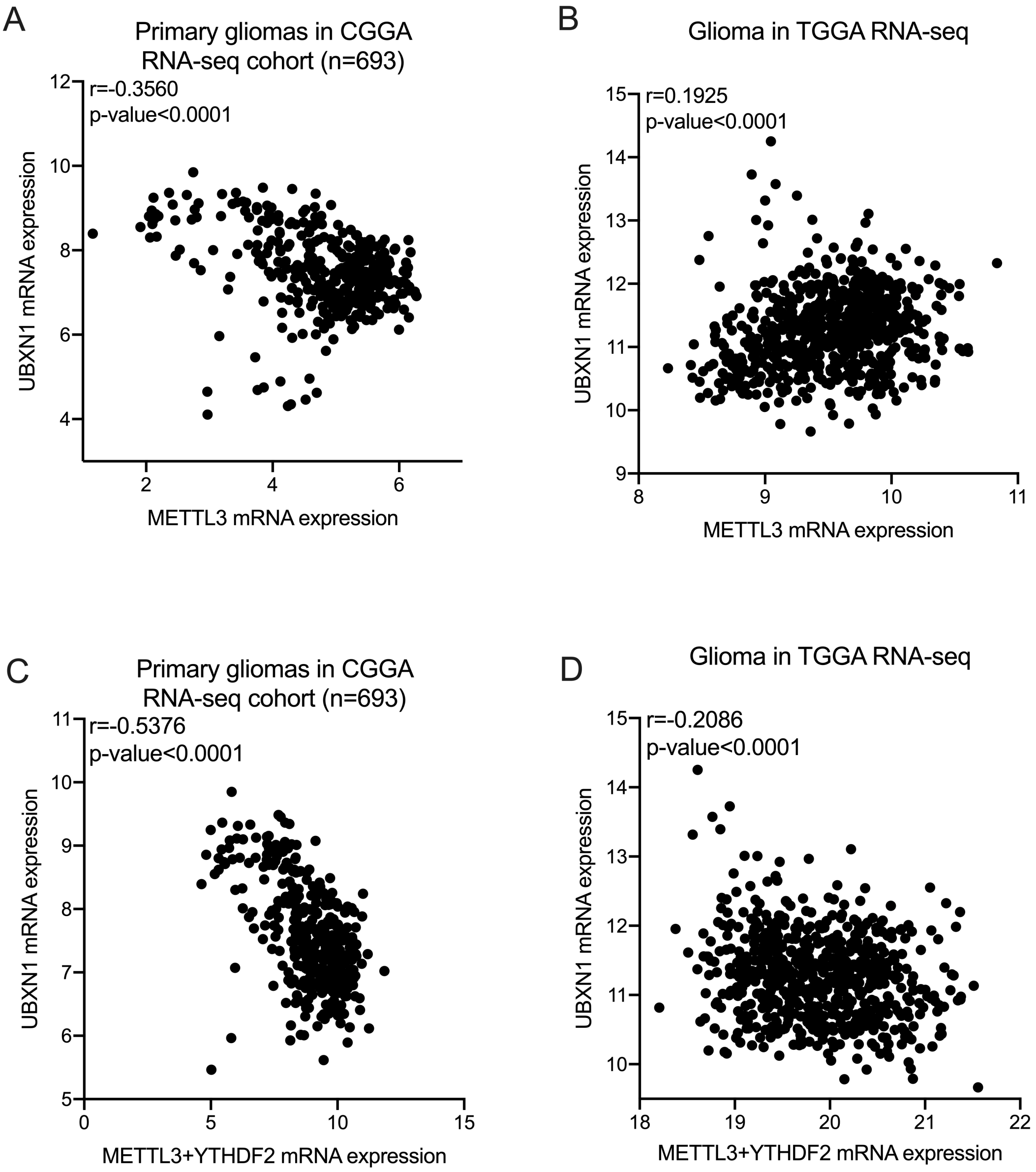

(A-B) The correlation analysis between UBXN1 and METTL3 mRNA expression levels. (C-D) The correlation analysis between UBXN1 and METTL3+YTHDF2 mRNA expression levels. r, Pearson r value.
